# A phenome-wide comparative analysis of genetic discordance between obesity and type 2 diabetes

**DOI:** 10.1038/s42255-022-00731-5

**Published:** 2023-01-26

**Authors:** Daniel E. Coral, Juan Fernandez-Tajes, Neli Tsereteli, Hugo Pomares-Millan, Hugo Fitipaldi, Pascal M. Mutie, Naeimeh Atabaki-Pasdar, Sebastian Kalamajski, Alaitz Poveda, Tyne W. Miller-Fleming, Xue Zhong, Giuseppe N. Giordano, Ewan R. Pearson, Nancy J. Cox, Paul W. Franks

**Affiliations:** 1 Genetic and Molecular Epidemiology Unit, Lund University Diabetes Centre, Department of Clinical Science, Lund University, Skåne University Hospital, Malmö, Sweden; 2 Oxford Centre for Diabetes, Endocrinology and Metabolism, University of Oxford, Oxford, UK; 3 Vanderbilt Genetics Institute, Vanderbilt University Medical Center, Nashville, TN USA; 4 Population Health and Genomics, University of Dundee, Dundee, UK; 5 Harvard T.H. Chan School of Public Health, Boston, MA USA

**Keywords:** Genetic variation, Type 2 diabetes, Obesity, Machine learning, Metabolism

## Abstract

Obesity and type 2 diabetes are causally related, yet there is considerable heterogeneity in the consequences of both conditions and the mechanisms of action are poorly defined. Here we show a genetic-driven approach defining two obesity profiles that convey highly concordant and discordant diabetogenic effects. We annotate and then compare association signals for these profiles across clinical and molecular phenotypic layers. Key differences are identified in a wide range of traits, including cardiovascular mortality, fat distribution, liver metabolism, blood pressure, specific lipid fractions and blood levels of proteins involved in extracellular matrix remodelling. We find marginal differences in abundance of Bacteroidetes and Firmicutes bacteria in the gut. Instrumental analyses reveal prominent causal roles for waist-to-hip ratio, blood pressure and cholesterol content of high-density lipoprotein particles in the development of diabetes in obesity. We prioritize 17 genes from the discordant signature that convey protection against type 2 diabetes in obesity, which may represent logical targets for precision medicine approaches.

## Abstract (web summary)

Coral et al. characterize genetically determined discordance between obesity and type 2 diabetes, identifying discordant genes that may convey protection against type 2 diabetes in obesity.

## Main

Cardiometabolic diseases are the leading cause of death globally, with obesity and type 2 diabetes mellitus (T2D) accounting for a large proportion of this burden^1^. The prevalences of obesity and T2D have risen sharply over the past decades worldwide^2^, corresponding with a shift to sedentary lifestyles and poor diet^3^. Even though obesity and T2D often coincide, their relationship is complex and remains incompletely understood. Indeed, while more than 80% of people with T2D also have obesity, 10–30% of people with obesity appear metabolically healthy^4–6^. Conversely, metabolic abnormalities occur in ~30% of normal-weight individuals^7–9^. Likewise, despite weight loss improving glycaemic control in people with T2D^10^, when T2D occurs in people with normal weight, mortality rates are higher than those in people with overweight or obesity^11,12^. Here, we refer to these divergent features as ‘discordant diabesity’. We focus on this unusual phenotype because it helps leverage the independent roles of excess adiposity and T2D in life-threatening disease.

To some extent, this discordance can be attributed to the imprecision with which body mass index (BMI), the conventional metric used to define obesity, characterizes adiposity^13,14^. For instance, even when BMI is comparable, lean and fat mass distributions often vary from one person to the next^15^. Genetics has helped provide pathophysiological explanations for discordant diabesity, whereby, collectively, common variants affecting adipose distribution mimic monogenic syndromes such as familial lipodystrophies^16–21^. Expanding our knowledge of the phenotypic signature of discordant diabesity using the quantitative framework of genetics may help elucidate the mechanisms by which the broader health consequences of excess adiposity varies from one person to the next.

Here, we characterize genetically determined discordant diabesity through a comparative analysis with its concordant counterpart (that is, where higher genetic risk of obesity and T2D coincide). We used a range of machine learning methods to undertake phenome-wide scans to identify traits other than T2D that distinctively characterize these profiles. We concluded by undertaking robust causal inference analyses to determine the causal relationships underlying discordant diabesity with other features of health and disease.

## Results

### Assembly of concordant and discordant diabesity profiles

**Extended Data Fig. 1:**
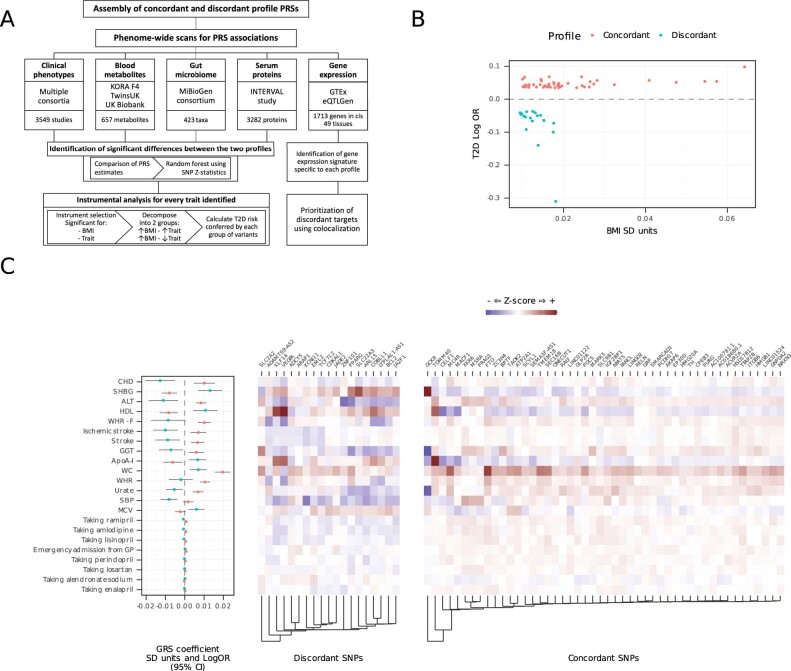
Analysis flowchart and profile identification. Panel A: Analysis flowchart. Panel B: BMI and T2D risk estimates of concordant and discordant SNPs after alignment to the BMI increasing allele. Panel C: summary-based concordant and discordant GRS coefficients (standard deviation units for continuous traits, log OR for binary traits). Traits shown have at least 1 estimate significant after 5% FDR correction and the difference between profiles is also significant after 5% FDR. Statistical tests were based on a Z-distribution and were two-sided. Bars show 95% confidence intervals. Sample sizes vary for every trait (> 100.000 for all traits). The heatmap shows the Z-scores of the SNPs in every trait, with the single-linkage tree at the bottom, separately for concordant and discordant SNPs.

An analysis flowchart is presented in Extended Data Fig. 1a. We first identified genetic instruments for BMI^22^ and T2D^23^ by cross-referencing publicly accessible genome-wide association study (GWAS) summary statistics, finding 67 relatively independent single nucleotide polymorphisms (SNPs) strongly associated with both conditions (*P* < 5 × 10^−8^). After alignment to the BMI-increasing allele, these variants were labelled as ‘concordant’ (48 SNPs) or ‘discordant’ (19 SNPs) according to the positive or negative sign of their coefficients for T2D, respectively (Extended Data Fig. 1b and Supplementary Table 1; replication shown in Supplementary Table 2). Visual inspection of correlation patterns between BMI and T2D signals at each locus was undertaken using regional association plots (Supplementary Figs. 1 and 2).

### Phenome-wide scans

**Fig. 1:**
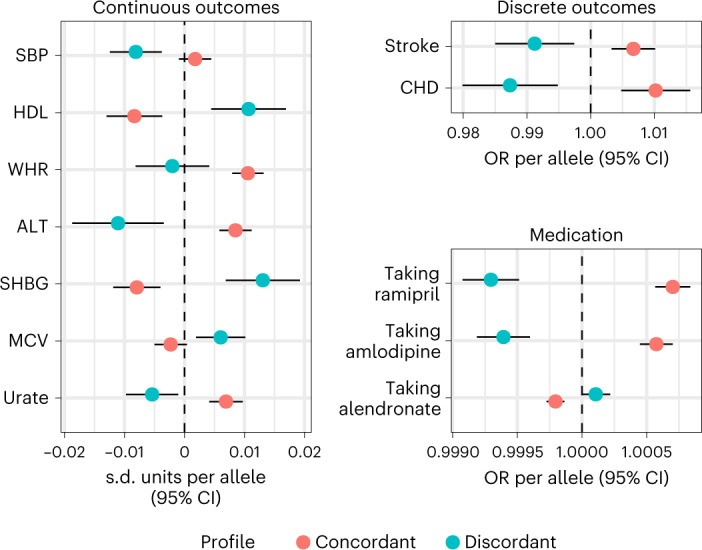
Summary-based comparison of concordant and discordant profiles. Concordant and discordant GRS coefficients for traits where we found differences between profiles using GWAS summary data. All are per-allele effect sizes, in s.d. units for continuous outcomes and ORs for binary traits (diseases and self-reported medication). Traits shown had at least one estimate significant after 5% FDR correction and the difference between profiles was also significant after 5% FDR. Statistical tests were based on a *z*-distribution and were two-sided. Bars show 95% CIs. Sample sizes vary for every trait (*N* > 100,000 for all traits).

Among the clinical phenotypes, we found that concordant and discordant diabesity profiles differed predominantly in cardiometabolic features including high-density lipoprotein (HDL) cholesterol, waist-to-hip ratio (WHR), waist circumference, and blood pressure (Fig. 1 and Supplementary Table 3). Generally, the discordant profile was associated with a favourable phenotypic signature compared to the concordant profile. For example, systolic blood pressure (SBP) was lower in the discordant compared to the concordant profile (SBP: *β*_C_ = 0.002 s.d. units per allele (95% confidence interval (CI): −0.001, 0.004), *β*_D_ = −0.008 s.d. units per allele (95% CI: −0.012, −0.004), pδ = 1.39 × 10^−4^). We also found differences in risk of coronary heart disease (CHD) and stroke, which were lower in the discordant compared to the concordant profile (for example, CHD: odds ratio (OR)_c_ = 1.01 per allele (95% CI: 1.01, 1.02), OR_D_ = 0.98 per allele (95% CI: 0.97, 0.99), pδ = 1.3 × 10^−6^). The levels of biomarkers of liver function such as gamma-glutamyl transferase (GGT) and alanine aminotransferase (ALT) enzymes were lower in the discordant relative to the concordant profile (for example, ALT: *β*_C_ = 0.008 s.d. units per allele (95% CI: 0.006, 0.011), *β*_D_ = −0.011 (95% CI: −0.019, −0.003), pδ = 2.07 × 10^−6^). SHBG, a protein also produced in the liver, was higher in the discordant as opposed to the concordant profile (*β*_C_ = −0.008 s.d. units per allele (95% CI: −0.012, −0.004), *β*_D_ = 0.013 s.d. units per allele (95% CI: 0.007, 0.019), pδ = 1.94 × 10^−8^). Additionally, the discordant profile was associated with higher mean corpuscular volume (*β*_C_ = −0.002 s.d. units per allele (95% CI: −0.005, 0), *β*_D_ = 0.006 s.d. units per allele (95% CI: 0.002, 0.01), pδ = 8.76 × 10^−4^) and lower levels of urate (*β*_C_ = 0.007 s.d. units per allele (95% CI: 0.004, 0.01), *β*_D_ = −0.005 s.d. units per allele (95% CI: −0.01, −0.001), pδ = 3 × 10^−6^) compared to the concordant profile. The odds of receiving treatment with alendronate was higher in the discordant than in the concordant profile, a drug indicated for osteoporosis (OR_C_ = 0.99 per allele (95% CI: 0.99, 0.99), OR_D_ = 1.001 per allele (95% CI: 1.001, 1.001), pδ = 3.26 × 10^−6^).

### Profile decomposition

Further exploration of the molecular features of the discordant and concordant profiles revealed that some variants used to characterize these profiles deviated from the overall pattern of trait association for their respective SNP set. Using single-linkage clustering on the SNP–trait matrix (Extended Data Fig. 1c), we identified two outliers in the concordant profile, one near *GCKR*, associated with higher SHBG and lower liver enzymes (SHBG: 0.07 (95% CI: 0.07, 0.08), *P* = 7.5 × 10^−199^) and a second near *TOMM40* associated with higher HDL (0.07 s.d. units per allele (95% CI: 0.06, 0.08), *P* = 3.7 × 10^−107^). In the discordant profile, the last variant to be aggregated to the clustering tree (that is, the SNP most distal from the other SNPs within its set) is located near *SLC2A2* and, in contrast to the overall discordant estimates, was associated with higher levels of AST and GGT (GGT: 0.02 s.d. units per allele (95% CI: 0.02, 0.03), *P* = 2.7 × 10^−22^).

### External validation in BioVU

**Fig. 2:**
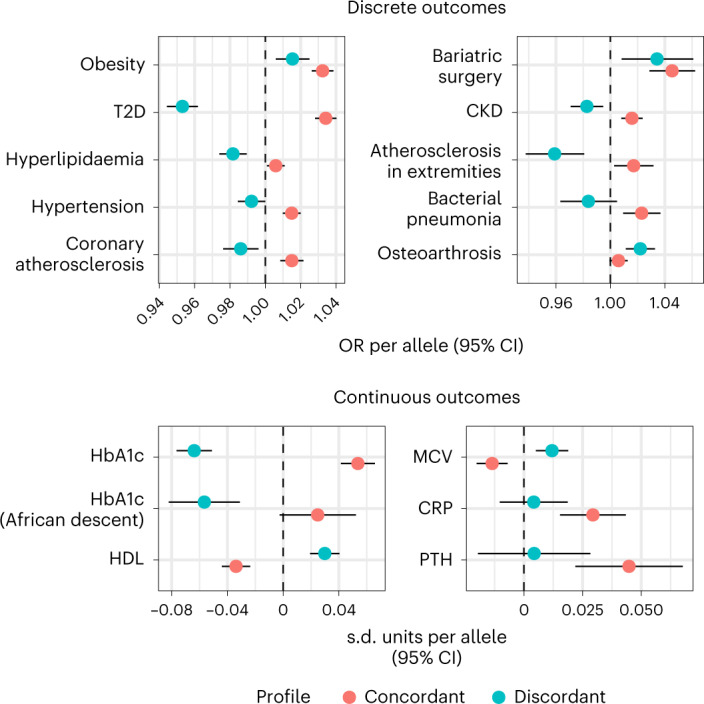
Comparison of concordant and discordant profiles in BioVU. Concordant and discordant GRS coefficients for traits where we found differences between profiles in BioVU. Analyses of disease endpoints included data for up to 48,544 individuals. Continuous outcomes included data for up to 68,724 and 13,661 individuals of European and African descent, respectively. All are per-allele effect sizes, in s.d. units for continuous outcomes and ORs for disease endpoints. Traits shown had at least one estimate significant after 5% FDR correction and the difference between profiles was also significant after 5% FDR. Statistical tests were based on a *z*-distribution and were two-sided. Bars show 95% CIs. CRP, C-reactive protein.

We sought replication of the discoveries outlined above in an independent European-ancestry cohort from BioVU, a de-identified collection of electronic health records and a linked biobank including inpatient and outpatient data from Vanderbilt University Medical Center (VUMC), a tertiary-care centre in Nashville, Tennessee, USA^24–26^. We constructed separate genetic risk score (GRS) coefficients for concordant and discordant profiles and assessed their association with multiple phenotypes (Fig. 2 and Supplementary Table 4). We first confirmed that the concordant and discordant GRSs were associated with higher obesity risk, respectively (OR_C_ = 1.03 per allele (95% CI: 1.03, 1.04), OR_D_ = 1.02 per allele (95% CI: 1.01, 1.02), pδ = 1.6 × 10^−3^) and that the concordant and discordant profiles were positively and negatively associated with diabetes diagnosis, respectively (OR_C_ = 1.03 per allele, (95% CI: 1.02, 1.04), OR_D_ = 0.95 per allele (95% CI: 0.94, 0.96), pδ = 3.2 × 10^−^^49^). Both scores were associated with increased odds of bariatric surgery (OR_C_ = 1.05 per allele (95% CI: 1.03, 1.06), OR_D_ = 1.03 per allele (95% CI: 1.008, 1.06), pδ = 0.24). We found divergent associations in multiple diseases directly related to the main traits (for example, essential hypertension (HT): OR_C_ = 1.014 per allele (95% CI: 1.009, 1.019), OR_D_ = 0.99 per allele (95% CI: 0.98, 0.99), pδ = 1.2 × 10^−6^). We also observed differences for other disease outcomes such as chronic kidney disease (OR_C_ = 1.02 per allele (95% CI: 1.01, 1.02), OR_D_ = 0.98 per allele (95% CI: 0.97, 0.99), pδ = 2.9 × 10^−6^) and osteoarthrosis (OR_C_ = 1.01 per allele (95% CI: 1, 1.01), OR_D_ = 1.02 per allele (95% CI: 1.01, 1.03), pδ = 0.012). Because both scores were also strongly associated with type 1 diabetes (T1D; OR_C_ = 1.05, (95% CI: 1.03, 1.05), OR_D_ = 0.96, (95% CI: 0.94, 0.97), pδ = 4.8 × 10^−17^), we repeated the analyses excluding individuals with T1D. This attenuated the differences between concordant and discordant profiles for a small subset of traits including diabetic retinopathy and end-stage chronic kidney disease.

We also assessed the association of each GRS to multiple laboratory measurements in individuals of European (*n* > 68,000) and African American (*n* > 14,000) descent (Supplementary Table 5). The value per individual was computed as the median value over all measurements after a quality-control pipeline described in detail elsewhere^27^. Significant differences were found for several glycaemic traits consistent with the diabetes risk profiles (for example, HbA1c: s.d. difference per concordant allele: 0.05 (95% CI: 0.04, 0.06), s.d. difference per discordant allele: −0.06 (95% CI: −0.08, −0.05), pδ = 1.5 × 10^−^^39^). We confirmed the difference between the two profiles in HDL and observed differences in the other two main lipid fractions (for example, triglycerides: s.d. difference per concordant allele: 0.02 (95% CI: 0.01, 0.03), s.d. difference per discordant allele: −0.04 (95% CI: −0.05, −0.03), pδ = 1.5 × 10^−^^14^). The findings for red blood cell phenotypes were also replicated, and additional differences were found in leucocyte count, urea, creatinine, phosphate, C-reactive protein and parathyroid hormone (PTH), all of which were higher in carriers of concordant SNPs. Of the liver enzymes, only ALT values were available for comparison, whose levels were weakly associated with the concordant but not the discordant GRS (s.d. difference per concordant allele: 0.14 (95% CI: 0.02, 0.26), s.d. difference per discordant allele: 0.06 (95% CI: −0.08, 0.2), pδ = 0.2). In individuals of African American descent, significant differences were found in HbA1c, glucose and urea levels in urine.

### Differences in mortality in UK Biobank

We examined the relationship of GRSs to mortality owing to cardiovascular events in >337,000 participants of European descent from the UK Biobank (mean follow-up of 11.8 years). Around 35,000 deaths were reported, of which approximately 20% were related to cardiovascular events. The concordant GRS was associated with higher mortality (hazard ratio (HR) per allele: 1.01 (95% CI: 1.01, 1.02)), whereas the discordant GRS was not (HR per allele: 0.99 (95% CI: 0.98, 1.01), pδ = 0.02). However, when assessing each SNP separately, we observed that the concordant variant near *TOMM40* was associated with lower incidence of cardiovascular mortality (HR per allele: 0.85 (95% CI: 0.81, 0.90), *P* = 4.54 × 10^−9^ and Supplementary Table 6).

### Differences in serum metabolites

**Fig. 3:**
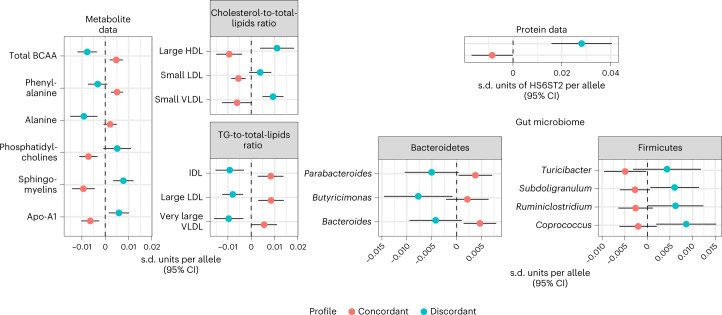
Comparison of concordant and discordant profiles in molecular phenotypes. Concordant and discordant GRS coefficients for traits where we found differences between profiles in molecular phenotypes. All are per-allele effect sizes, in s.d. units. Findings in metabolites shown here are derived from TwinsUK + KORA F4 (*N* = 7,824) and the UK Biobank (*N* = 115,078). Protein data were derived from the INTERVAL study (*N* = 3,301). Traits shown in these two domains had at least one estimate significant after 5% FDR correction, and the difference between profiles was also significant after 5% FDR. Statistical tests were based on a *z*-distribution and were two-sided. Bars show 95% CIs. Microbiome data came from the MiBioGen consortium (*N* = 18,340); the genii shown here had at least one estimate nominally significant, and the difference between estimates was also nominally significant (two-sided *P* < 0.05).

Of the metabolites available, those related to lipid subfractions were the strongest discriminators of concordant and discordant profiles (Fig. 3 and Supplementary Table 7). Discordant diabesity was associated with higher cholesterol in lipoprotein particles of all densities, while lower triglyceride content in lipoprotein particles of low densities, as opposed to concordant diabesity (for example, free cholesterol in HDL: *β*_C_ = −0.008 s.d. units per allele (95% CI: −0.01, −0.005), *β*_D_ = 0.008 s.d. units per allele (95% CI: 0.004, 0.013), pδ = 3.09 × 10^−10^). Discordant diabesity also correlated with lower levels of branched-chain amino acids and aromatic amino acids, whereas in concordant diabesity they tended to be higher (total concentration of branched-chain amino acids: *β*_C_ = 0.004 s.d. units per allele (95% CI: 0.002, 0.008), *β*_D_ = −0.008 s.d. units per allele (95% CI: −0.012, −0.003), pδ = 1.46 × 10^−6^).

### Differences in gut microbiota

There were no differences between pooled concordant and discordant estimates for bacterial abundance in the gut that were statistically significant after false discovery rate (FDR) correction. Across ten taxa, several were nominally associated (*P* < 0.05) within either the concordant or the discordant profiles (Fig. 3 and Supplementary Table 8). Four of these belonged to the phylum Bacteroidetes (family Bacteroidaceae and geni *Bacteroides*, *Parabacteroides* and *Butyricimonas*), all of which were less abundant in discordant relative to concordant diabesity (for example, family Bacteroidaceae: *β*_C_ = 0.005 s.d. units per allele (95% CI: 0.001, 0.008), *β*_D_ = −0.004 s.d. units per allele (95% CI: −0.004, −0.01), pδ = 0.004). The remaining taxa belonged to the phylum Firmicutes, most of them members of the obligately anaerobic class Clostridia, which tended to be more abundant in the discordant profile compared to the concordant profile (for example, genus *Subdoligranulum*: *β*_C_ = −0.003 s.d. units per allele (95% CI: −0.006, 0.001), *β*_D_ = 0.006 s.d. units per allele (95% CI: 0.007, 0.011), pδ = 0.006). The family Lactobacillaceae was also lower in the discordant compared to the concordant profile (*β*_C_ = 0.006 s.d. units per allele (95% CI: 0.001, 0.01), *β*_D_ = −0.006 s.d. units per allele (95% CI: −0.014, 0.003), pδ = 0.02).

### Differences in serum protein levels

We found a significant difference between concordant and discordant estimates after FDR correction in a single protein: heparan sulfate 6-*O*-sulfotransferase 2 (HS6ST2), which was higher in discordant relative to concordant diabesity (*β*_C_ = −0.01 s.d. units per allele (95% CI: −0.017, 0), *β*_D_ = 0.03 s.d. units per allele (95% CI: 0.02, 0.04), pδ = 7.52 × 10^−7^; Fig. 3). These analyses may be underpowered given that the effect of variants in *trans* is likely to be weaker than that of those in the gene encoding the protein. Thus, we also searched for strong *cis* effects (*P* < 5 × 10^−8^) in the discordant profile. We found one association between the discordant variant near *PPARG* and metalloproteinase inhibitor 4 (TIMP4; *β* = −0.28 s.d. units per allele (95% CI: −0.35, −0.2), *P* = 5 × 10^−14^).

### Functional annotation using DEPICT

We used the Data-driven Expression Prioritized Integration for Complex Traits (DEPICT)^28^ tool to compare the enrichment for tissues and biological pathways in each profile (Supplementary Figs. 3–5). The most notable difference was the significant enrichment (*P* < 0.05) for adipose tissue in the discordant profile, which was not found in the concordant profile. We also found significant enrichment for adrenal glands, ileum and kidney in the discordant but not in the concordant profile. Conversely, there was significant enrichment for endocrine tissue and retina in the concordant but not the discordant profile. Tissues for which there was significant enrichment in both profiles included pancreas and myometrium.

### Gene expression and splicing in discordant diabesity

We found 506 genes whose expression/splicing was significantly influenced by concordant SNPs and 76 which were influenced by discordant SNPs across multiple tissues in GTEx^29^. In eQTLGen^30^, we found significant associations of concordant SNPs with expression of 493 genes. Discordant SNPs were associated with 94 genes. Around 46% of all the associations found in GTEx were replicated in eQTLGen (47% of the genes associated with concordant SNPs; 39% of the genes associated with discordant SNPs).

**Fig. 4:**
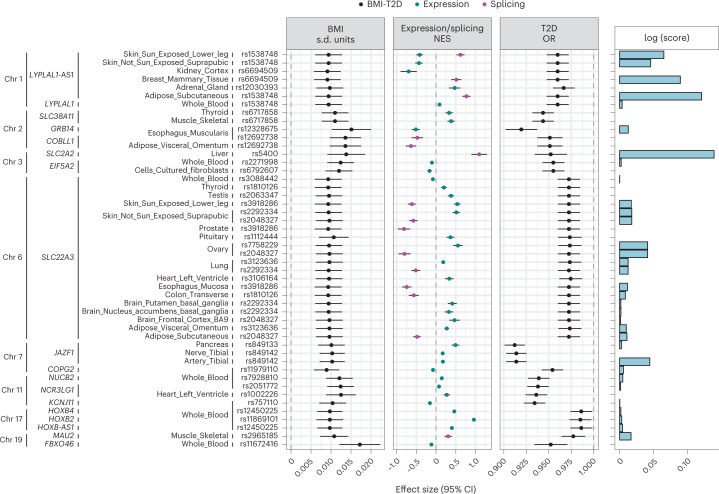
Genes with likely discordant pleiotropic effects on BMI and T2D. Genes with likely pleiotropic, yet discordant, effects on BMI and T2D, as found in the SMR & HEIDI analysis. Genes were sorted by their chromosome location and tissue where the pleiotropic association was found, as well as the lead expression quantitative trait loci (eQTL). The first three panels comprise the effect sizes of the lead eQTL on BMI (s.d. units), gene expression/splicing (normalized effect size) and T2D risk (OR), respectively. Bars represent 95% CIs. The right panel represents the logarithm of the tissue-of-action score. BMI data were derived from the GIANT + UK Biobank meta-analysis (*N* = 681,275). Gene expression data came from the GTEx (*N* = 838) and eQTLGen consortia (*N* = 31,684). T2D data came from the DIAGRAM meta-analysis dataset (*N* = 158,186).

To identify genes most likely involved in the molecular mechanisms leading to discordant diabesity, we chose genetic instruments for the 76 genes whose expression was influenced by discordant SNPs in the corresponding tissues in GTEx, and for the 94 genes in eQTLGen. A prerequisite for these instruments was that they are strongly associated with BMI (*P* < 5 × 10^−8^). We followed the SMR & HEIDI approach^31^, which utilizes the strongest instrument for gene expression/splicing within the *cis* region of the corresponding gene (±500 Mb from the transcription start site) to calculate an estimate of the pleiotropic association across gene expression, BMI and T2D risk. This approach then determines if the association found reflects true pleiotropy rather than mere linkage by testing for heterogeneity of the estimates of SNPs in linkage disequilibrium (LD) with the lead SNP. We found 17 genes with robust expression signals for obesity and T2D whose directions of effect were in contrast (FDR-corrected *P* < 5%, p_HEIDI_ > 0.01; Fig. 4 and Supplementary Table 9). To locate the most likely tissue of action for these genes, we followed a scoring procedure^32^ through which a tissue specificity score is derived for each gene. This is calculated as (i) the proportion of median expression (in transcripts per million) across specific tissue types catalogued in GTEx and (ii) evidence of promoter/enhancer histone marks surrounding the genetic instruments, derived from multiple cell lines classified anatomically by the RoadMap Epigenomics Project^33^ that could be mapped to tissue samples in GTEx. For each gene, we sorted tissues where we found pleiotropic links according to its specificity score and presence of promoter/enhancer signals for the genetic instrument. This allowed us to prioritize potential main action sites of relevance to discordant diabesity, for example, *LYPLAL1* in adipose tissue and *JAZF1* in vasculature and pancreas, while confirming the widespread effects of *SLC22A3* across multiple organs.

### Discordant diabesity genes as therapeutic targets

We performed a lookup of the genes identified previously in the comprehensive public access databases DGIdb^34^ and PHAROS^35^. Notably, there was evidence of interaction between three of the genes with strong pleiotropic associations (*SLC2A2*, *SLC22A3* and *KCNJ11*) and metformin in both databases. *SLC22A3* interacted with various quinoline derivatives (decynium-22, disprocynium-24, found in both databases), SarCNU (an antineoplastic drug in phase 2 clinical trials), derivatives of the alpha blocker phenoxybenzamine, corticosterone and colchicine. There is also evidence of potent inhibition of GLUT2, the protein product of *SLC2A2*, by a specific class of pyrazolopyrimidines. *SLC38A11*, *MAU2* and *FBXO46* are classified in the ‘Tdark’ level of target development in the PHAROS database, composed of understudied targets, while the remaining genes fall under the ‘Tbio’ level, which includes targets with no known interactions yet satisfying other conditions, such as having functional annotations based on experimental evidence, repeated mentions in publications indexed in PubMed, and 50 or more available commercial antibodies.

### Instrumental variable analyses

To quantify the potential impact of traits that emerged from the previous steps on offsetting the diabetogenic effect of obesity, we derived genetic instruments for each of these traits using SNPs that were also robustly associated with BMI (*P* < 5 × 10^−8^) and decomposed these instruments into two groups based on their direction of effect on the trait of interest after alignment to the BMI-increasing allele. We then constructed two GRSs, one for each group of variants, and calculated the T2D risk conferred by each GRS using summary data from the DIAGRAM consortium; we focused on GRSs that confer protection from T2D.

**Extended Data Fig. 2:**
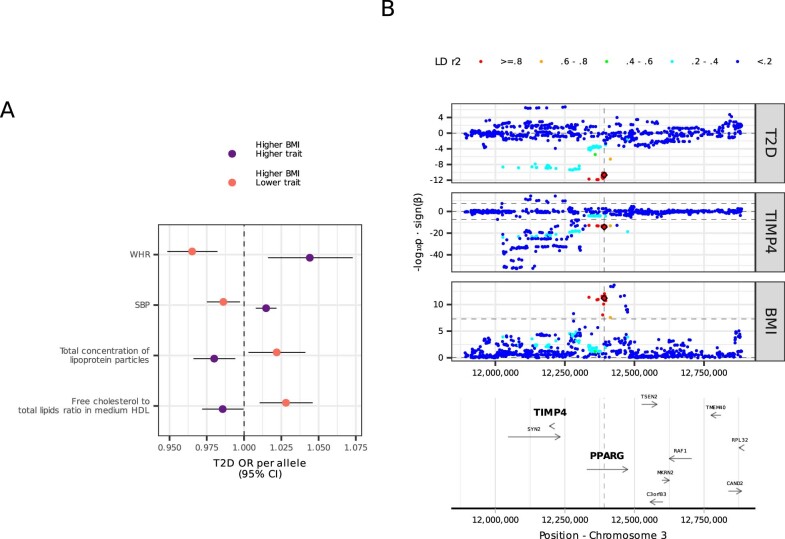
Traits with potential causal effect on diabesity discordance. Panel A: Traits where a difference was found in the comparison of profiles and one of the two direction-specific GRS associated with BMI was associated with lower risk of T2D (two-sided Z-statistic P < 0.05). To derive the GRS we used BMI data from the GIANT + UK Biobank meta-analysis (N = 681,275). WHR data came from the GIANT consortium (N = 212,244). SBP data came from the meta-analysis performed by the ICBP (N = 757,601). Metabolite data came from the UK Biobank (N = 115,078). Estimates represent T2D OR, bars represent 95% confidence intervals, which are derived from the DIAGRAM meta-analysis (N = 158,186). Panel C: Regional association plot showing the pleiotropic effect of genetic instruments for blood levels of TIMP4 protein and high BMI and lower T2D risk. Protein data was derived from the INTERVAL study (N = 3,301).

From the clinical phenotypes, the GRSs that conveyed higher BMI but lower WHR and SBP were significantly associated with lower T2D risk (Extended Data Fig. 2a and Supplementary Table 10). For example, the estimate for the GRS associated with higher BMI but lower WHR had an OR of 0.96 per allele (95% CI: 0.94–0.98, *P* = 6.71 × 10^−5^). Some traits in the clinical phenotypes required instruments to be in *cis* with the gene encoding the corresponding protein (for example, SHBG), to prevent confounding due to pleiotropy. We found two such instruments for ApoA1 and SHBG, respectively, which were not associated with T2D risk (*P* = 0.17 and 0.84, respectively; Supplementary Table 11) despite their strong association with higher BMI. From the analysis of metabolites, we found two GRS coefficients associated with higher BMI and lower T2D risk. The strongest protection was found for the GRS conferring higher total concentration of lipoprotein particles (OR: 0.98, 95% CI: 0.96, 0.99, *P* = 0.006; Supplementary Table 12), consistent with our findings in the phenome scans.

To test for the potential causal effect on diabesity discordance of HS6ST2 and TIMP4, the two proteins identified in the previous analysis, we searched for valid instruments (*P* value for both protein levels and BMI < 5 × 10^−8^) in the *cis* region of the corresponding genes. We could only derive a valid instrument for TIMP4. Using the SMR & HEIDI method, we found a significant pleiotropic effect (*P* = 3.8 × 10^−7^, p_HEIDI_ = 0.4; Extended Data Fig. 2b). However, we noted that the lead instrument and its closest proxies were located within *PPARG*, which is proximal to *TIMP4*.

No instruments for the microbial taxa where we found nominally significant differences reached the significance threshold required for BMI. Extending the exploration to other taxa revealed a single significant association (*P* < 5 × 10^−8^) of the A allele of rs1530559 (a variant within the lactase persistence haploblock^36^ in LD with the lactase functional variant rs4988235 (*r*^*2*^ = 0.4)) with higher BMI and lower abundance of the order Bifidobacteriales. This variant was not associated with T2D risk (*P* = 0.76).

### Individuals within the top decile for each profile

To determine the relevance of concordant and discordant profiles in people with obesity (≥30 kg/m^2^), we focused on this subgroup in UK Biobank who localized to the top decile of one of the two profile GRSs^37^. Consistent with a binomial distribution, 18% of individuals with obesity were present in the two groups of extreme GRSs. The health characteristics of these individuals differed from all others with obesity (Supplementary Table 13) in several ways: for example, HbA1c levels in individuals with obesity and the extreme concordant GRS were higher compared to all other individuals with obesity (Kruskal–Wallis *P* = 5.94 × 10^−12^). Conversely, individuals with obesity and an extreme discordant GRS had significantly lower HbA1c compared to the rest of individuals with obesity (*P* = 2.71 × 10^−42^). Persons with obesity at both extreme GRSs are also distinguished from the rest by the main clinical features identified in our previous analyses such as SBP, HDL and ALT. WHR did not adequately separate the concordant or discordant extreme GRS from the wider group of people with obesity. However, because the initial phenome scan revealed a gender-specific difference in WHR between concordant and discordant profiles, (Extended Data Fig. 1c), we performed an additional analysis for WHR stratified by sex, where we found that women with extreme discordant GRS had significantly lower WHR compared to other women with obesity (*P* = 6.05 × 10^−10^).

### Comparison with previous studies of discordant diabesity

We compared our results to those obtained in three previous investigations of discordant variants. For instance, Mahajan et al.^23^ calculated the change in estimates of SNPs associated with T2D before and after adjustment for BMI. They found 15 loci where signals were enhanced after adjustment, which was attributed to discordance. Consistently, the SNP effects in the diabesity discordant profile derived here were enhanced, while those of the concordant profile were attenuated after adjustment (as described in ref. ^38^; Supplementary Table 14). The change in SNP effect estimates was consistently associated with SNP effects on BMI (*R*^2^ = 0.8; Supplementary Fig. 6). However, we observed that for four of the 19 SNPs (20%) from the discordant set near *PPARG*, *JAZF1*, *KCNJ11* and *LYPLAL1*, the change in SNP effect estimates was less than predicted. These discordant variants are those most likely to directly alter the relationship between BMI and T2D. This is consistent with the known effect of *PPARG* on adipocyte differentiation, and with our findings linking adipose tissue-specific gene expression at the *LYPLAL1* locus with higher BMI but lower T2D risk. Similarly, we found that *KCNJ11* and *JAZF1* had discordant effects on BMI and T2D, which is related to tissue-specific expression in heart and arteries; variants at both loci are known to influence insulin secretion.

We also sought replication of a finding from Pigeyre et al.^27^ linking discordance to levels of the protein IGFBP-3 in blood. We were not able to replicate this finding (Supplementary Table 15), possibly due to differences in the characteristics of the cohorts where this relationship was found. For our analysis, we used summary data from the INTERVAL study^39^, which includes predominantly healthy blood donors of European ancestry. In contrast, Pigeyre et al. used data from the ORIGIN trial, a cohort composed of individuals of European (47%) and Latin American (53%) ancestries, enriched for T2D cases (>80% had a prior diagnosis).

Finally, we searched for the SNPs comprising the concordant and discordant profiles described above in the cluster analysis of discordant SNPs performed by Huang et al.^21^. Fourteen of the 19 discordant SNPs identified in our analysis (78%) are among or in LD with the 62 SNPs identified by Huang et al (*r*^2^ > 0.1 within a 1-Mb window, as specified in the publication; Supplementary Table 16). Two of the subclusters described by the authors were significantly overrepresented by these 14 SNPs: 5 (*ARAP1*, *ADCY5*, *PPARG*, *TCF7L2*, *KCNJ11-NCR3LG1*) were in the subcluster characterized mainly by higher BMI and lower fasting glucose and risk of T2D (enrichment *P* = 1.6 × 10^−^^3^) and 4 (*GRB14*, *LYPLAL1*, *ADAMTS9* and *VEGFA*) in the subcluster that conveyed an apparent protective effect on multiple cardiometabolic traits via peripheral adipose distribution (higher BMI and body fat percentage, and lower WHR; enrichment *P* = 0.04). Four concordant variants (at *GCKR, TOMM40, AKAP6* and *PPP1R3B-TNKS-MSRA*) were also among the 62 SNPs described by Huang et al. As opposed to other variants in the concordant set, the variant in *AKAP6* was associated with lower SBP (in ICBP GWAS: *β* = −0.25 mm Hg (95% CI: −0.38, −0.12), *P* = 1.25 × 10^−4^) and the variant near *PPP1R3B-TNKS-MSRA* was associated with higher HDL (*β* = 0.02 s.d. units (95% CI: 0.012, 0.027), *P* = 1.72 × 10^−6^). As we found and discussed in our analyses, *TOMM40* and *GCKR* deviate from the concordant set owing to their favourable associations with lipids and liver enzymes that resemble the discordant set, a pattern that was also reported by Huang et al.

## Discussion

Obesity conveys heterogenous effects in cardiometabolic health, making disease prevention and management challenging. Here we used genetics to deconstruct the obesity phenotype into concordant and discordant diabesity, with strikingly different health characteristics beyond diabetes and obesity. Through transcriptomic, metabolomic and metagenomic analyses, we identified biomarkers that shed light on mechanisms of action and may aid risk stratification. Further analyses identified potential targets for drug development and drug repurposing.

Obesity and T2D often coalesce, owing largely to the mediating effect of peripheral insulin resistance caused by excess adiposity. The trait discordances described here reflect mechanisms involved in uncoupling obesity risk from T2D risk, thereby exposing diabetes-independent pathways through which obesity affects disease risk, for example, through adipose distribution. It is likely that both a higher capacity to expand adipose tissue in the gluteo-femoral compartment^40,41^ and lower abdominal region around organs such as the liver, which might underlie the difference seen in biomarkers of liver failure^42^, play important and independent roles in genetically determined diabesity discordance.

Another key phenotypic distinction between concordant and discordant profiles concerns blood pressure. Although T2D often causes vascular dysfunction, changes in the vascular bed may also precede metabolic perturbations through nutrient and hormonal flux^43,44^, affecting pancreas, muscle and adipose tissue^45^. For instance, capillary recruitment and permeability are key determinants of whole-body glucose uptake and glycaemic variation^46^.

Our findings relating to lipid metabolites support the use of more refined profiling of lipid subfractions to help determine risk in people with obesity. The cholesterol content of HDL particles and BCAA levels appear especially informative biomarkers^47^, possibly because they enhance glucose homeostasis in obesity by improving cross-talk between peripheral tissue and the liver^48^.

Despite the contrasting health consequences of the two diabesity profiles, bariatric surgery was equally likely, which may predispose one group to health benefits following surgery, whereas the other may not benefit in this way.

We found a significant difference between concordant and discordant profiles in levels of HS6ST2, a protein expressed in brain, kidney and ovaries, which in animal knock-out models shows a strong association with increased body weight and insulin resistance, possibly owing to enhanced adipocyte differentiation^49,50^. We found only one robust pleiotropic effect for discordant diabesity at *TIMP4*, which is proximal to *PPARG*, the likely causal gene. Moreover, PPARG activator medication inhibits matrix metalloproteinases^51,52^. *TIMP4* has been associated with adipogenesis, possibly through its effect on the adipose tissue extracellular matrix in obesity^53^.

The colocalization analyses underscore the importance of tissue pleiotropy and tissue cross-talk in the molecular mechanisms of diabesity discordance. This is especially evident for *SLC22A3*, but also for other potential targets such as *LYPLAL1*, whose differential expression in both adipose tissue and adrenal glands appears linked to discordant diabesity. Moreover, three of the genes with pleiotropic links to T2D risk (*SLC2A2, SLC22A3* and *KCNJ11*) interact with metformin. This suggests a potential effect of metformin in shifting individuals with obesity from a concordant to a discordant phenotype. *SLC2A2* encodes GLUT2, which is part of the glucose sensor apparatus in pancreas and liver and is involved in intestinal glucose absorption in the gut^54^. Variants in this gene have been associated with preference for sugary foods^55^ and modified response to metformin^56,57^. *SLC22A3* encodes OCT3, a protein widely expressed across tissues that aids adipocyte beiging^58^ and perivascular adipose tissue remodelling^59^. *KCNJ11* encodes the Kir6.2 subunit of the ATP-sensitive potassium channel. As this is the target of sulphonylureas, this group of drugs may also harbor potential candidates for the phenotype shift to discordance in diabesity. The other ligands identified in the lookup may also constitute potential therapeutic agents to prevent cardiometabolic complications in obesity. For the rest of the genes, especially those in the ‘Tdark’ level in PHAROS, follow-up functional experiments in the tissues indicated by the lead genetic instruments and its corresponding epigenetic annotations are warranted.

Certain SNPs deviate from the overall association pattern of the profile within which they reside. In the concordant profile, the BMI-increasing allele of the variant near *TOMM40* increases T2D risk but, unlike other SNPs in the same profile, is associated with a better lipid profile and lower cardiovascular disease mortality. This gene and others in its proximity (*APOC1* and *APOE*) have been consistently implicated in lipid metabolism^60^. In the discordant profile, the variant *SLC2A2* conveys protection against T2D risk despite being associated with heavier weight and higher blood pressure, and worse liver function and dyslipidaemia. The opposite pattern was observed in the concordant variant in *GCKR*, which encodes a regulatory protein that inhibits glucokinase. This reflects disparate phenotypic effects of modulating the glucose sensor apparatus at different levels^54^. Deeper characterization of these mechanisms can further improve obesity stratification.

Although no statistically robust differences were observed in gut microbiota between the two diabesity profiles, possibly owing to low statistical power, nominal differences emerged in taxa belonging to the Bacteroidetes and Firmicutes phyla, which together constitute 90% of the human intestinal flora^61^. Our results indicate higher Firmicutes and lower Bacteroidetes abundance in discordant diabesity, which may result in enhanced production of short-chain fatty acid species such as butyrate, which is involved in glucose-lowering and anti-inflammatory mechanisms^62^.

Previous strategies to characterize the discordance between BMI and metabolic risk have been based on predefined sets of phenotypes traditionally linked with metabolic status^19,21^. Our phenome-wide approach consisted of leveraging the wealth of genetic associations harvested to date to dissect the phenotypic structure relevant for discordant diabesity, having three main advantages: (1) variables defining the differential phenotypic structure of each profile are selected a in data-driven manner across many phenotypic layers; (2) leveraging genetic data across multiple datasets enhances power and minimizes cohort-specific biases that would be anticipated if analyses were performed in a single cohort; and (3) although concordant and discordant diabesity profiles may be driven by molecular mechanisms that are independent of DNA variation, using germline DNA variants helps mitigate reverse causality and other sources of confounding that hamper the interpretation of associations for most other types of biological variation and phenotypes. An example of this is the analysis of epigenetic factors, which has led to identification of obesity sub-phenotypes even in the context of genetic homogeneity, as found in monozygotic twins that are discordant for adiposity traits^63^. However, these findings might be driven by variations in environmental exposures and behaviours that exist within and between twin pairs, as well as confounded by factors such as age, which differed between twin pairs in the reported analyses.

In conclusion, obesity profiles with either diabetogenic or antidiabetogenic proclivities reveal distinctive aetiological subtypes, with key differences in fat distribution, blood pressure and cholesterol content in HDL particles. We identified 1 protein (TIMP4) and 17 genes potentially involved in the molecular mechanisms leading to diabesity discordance, involving pleiotropic effects across multiple tissues.

## Methods

### Study populations

#### BioVU

Collection of electronic health records in BioVU was established in 1990 and includes data on billing codes from the International Classification of Diseases, 9th and 10th editions (ICD-9 and ICD-10). Disease phenotypes (‘phecodes’) are derived from these billing codes as described previously^24^ and case, control and exclusion criteria are defined. Two codes on different visit days were required to instantiate a case for each phecode. The biobank was launched in 2007 and comprises excess blood samples that their donors had consented for use in biomedical research. Details of programme operations, ethical considerations, continuing oversight and patient engagement are published elsewhere^25^. DNA samples were analysed using genome-wide genotyping platforms including Illumina multi-ethnic genotyping array. After quality assessment, the genotype data were then imputed to the Haplotype Reference Consortium reference panel at the Michigan imputation server. Populations of African American and European descent were identified by projecting individuals onto the major principal-component space derived from 1000 Genomes reference panel.

#### UK Biobank

The UK Biobank is an ongoing prospective study of approximately 500,000 adults. Initial enrolment took place from 2006 to 2010 and included individuals aged 40–69 years across the United Kingdom^64^. It has collected comprehensive genetic and phenotypic information, biochemical assays and longitudinal health outcomes through health records, such as hospitalization and mortality. The genotypes were assayed using the UK Biobank Lung Exome Variant Evaluation and the Applied Biosystems UK Biobank Axiom Array, and imputed to the Haplotype Reference Consortium panel. Population structure was also assessed using principal-component analysis. We excluded individuals with inconsistency between their reported and genetic sex, had sex chromosome aneuploidy or were outliers for heterozygosity or missingness. Only individuals who were included in the calculation of genetic principal components were included, which ensures minimal genetic kinship with other participants.

### Single-nucleotide polymorphism selection to construct concordant and discordant genetic profiles

We cross-referenced the largest GWAS for BMI and T2D and extracted common biallelic SNPs (minor allele frequency (MAF) > 1%). Insertions, deletions and potentially ambiguous palindromic SNPs (A/T or C/G alleles with MAF > 30%) were excluded. Because both GWAS were conducted predominantly in populations of European descent, we used 1000 Genomes EUR reference panel for clumping (*r*^2 ^< 0.01 over a 500-kb window) to identify nearly independent SNPs that were strongly associated with both conditions (*P* < 5 × 10^−8^). The directions of the effect of these SNPs on T2D were consistent in a second independent set of GWAS summary statistics extracted from the FinnGen database^65^ (Supplementary Table 2).

### Phenome-wide scans

We extracted association data for concordant and discordant SNPs from a variety of sources. From the curated repository Open GWAS^66^, which we queried using the ‘ieugwasr’ package in R, we gathered data for >3,500 traits derived from UK Biobank and other consortia for a variety of traits; these traits are termed ‘clinical phenotypes’. In cases where the effect of a SNP on a trait was not found, we looked for the effect of the nearest proxy SNP up to an *r*^2^ of 0.5 over a 500-kb window. We only kept estimates obtained from European ancestry populations in order to be consistent with the GWAS used to identify the genetic profiles. To prevent inclusion of inflated signals due to low sample size, we only included studies of more than 500 individuals; for binary traits, we required at least 25 minor alleles in the smallest group^67^. We calculated *z*-scores by dividing the *β* coefficients by their corresponding standard errors, and then we computed standardized effect sizes as a function of MAF and sample size *n* using equations (1) and (2) (ref. ^31^):

1 $$\mathrm{SE} = \frac{1}{{\sqrt {2 \times \mathrm{MAF} \times \left( {1 - \mathrm{MAF}} \right) \times (n + z^2)} }}$$

2 $$\beta = z \times \mathrm{SE}$$

We aligned all the estimates from these scans to the BMI-increasing alleles, so that they represent phenotypic variations associated with higher BMI in both profiles.

We also obtained data for 657 blood metabolites^68,69^ and 3,282 proteins in plasma^39^. Associations with several bacterial taxa in the gut were obtained from the MiBioGen consortium^70^. Association with expression and splicing of nearby genes in multiple tissue samples and in whole blood were obtained from data generated by GTEx and eQTLGen consortia, respectively.

### Profile comparison

We then compared the effects of discordant versus concordant SNPs for every trait in two stages: we first obtained the combined effect of concordant (*β*_C_) and discordant (*β*_D_) SNPs separately using a random-effects meta-analysis with the Paule–Mandel estimator of between-SNP variance τ^2^ (refs. ^71^^,72^). We then calculated their difference δ = |*β*_C_ − *β*_D_| and computed its standard error as $\mathrm{SE}_\delta ^2 = \mathrm{SE}_C^2 + \mathrm{SE}_D^2$^73^. We excluded from these analyses T2D traits (Supplementary Table 17). Traits in which any of the combined estimates *β*_C_ or *β*_D_ and δ were statistically significant after 5% FDR correction were taken forward to stage two, where we converted the effect estimates for each SNP and the selected traits to *z*-scores and placed them in a SNP–trait matrix, with SNPs coded as ‘0’ if concordant and ‘1’ if discordant. We then trained several Random Forest classifiers (1,000 iterations) to this matrix, which attempted to classify SNPs in their correct category, and used the Boruta algorithm^74^ to identify which traits were relevant to distinguish discordant from concordant SNPs. Briefly, this algorithm creates randomly shuffled copies of all traits in the SNP–trait matrix, and then evaluates for each trait if its contribution to the accuracy of decision trees in the Random Forest is higher than its corresponding random set.

### Genetic risk score analyses

Concordant and discordant GRSs for an individual *i* were calculated as:

3 $$\mathrm{PRS}_{Pi} = \mathop {\sum }\limits_{j \in P}^{M_P} G_{ij}$$

Where *P* is the set of *M*_*P*_ SNPs belonging to the concordant or discordant profiles and *G*_*ij*_ is the genotype for SNP *j* in individual *i*. In BioVU, association analyses were carried out for each GRS using R package ‘PheWAS’ (v0.99.5-5)^24^. We kept phecodes with at least 200 cases^67^ and identified those associated with either of the GRS coefficients and a significant difference between the estimated effects after a 5% FDR correction.

In the UK Biobank, we examined the relationship of GRSs to mortality due to cardiovascular events in individuals followed up to the latest censor date (30th September 2021) using Cox regression. Primary cause of death was ascertained using ICD-10 codes reported in death certificates (Supplementary Table 18). All association models were adjusted for age, sex and first ten genetic principal components.

### SMR & HEIDI

The SMR method consists of identifying for a protein or gene the strongest association signal, which is used as a genetic instrument to test for its pleiotropic effect on an outcome. The HEIDI method consists of calculating the heterogeneity in the estimates of SNPs in LD with the lead SNP used in SMR. A higher p_HEIDI_ value means heterogeneity is less likely, which supports true pleiotropy across the gene/protein and outcome signal, while a lower p_HEIDI_ value means there is heterogeneity in the estimates, and the SMR signal is probably due to linkage. We consider an association to be true pleiotropy if p_HEIDI_ > 0.01 (ref. ^75^). We retained signals where we found evidence of true pleiotropy for both BMI and T2D.

### Scoring method using epigenetic annotation

The scoring method to identify the most likely tissue of action assumes that if a genetic instrument for the expression of a gene in a certain tissue where it is highly expressed (that is, high tissue specificity) is in or close (in LD) to a promoter/enhancer region in the same tissue, and this genetic instrument is also associated with an outcome, then it is likely that the pleiotropic association on the outcome is due to perturbation of gene activity in that tissue. Promoter/enhancer signals were obtained by querying the RoadMap Epigenomics Project through the ‘haploR’ package in R.

### Genes as therapeutic targets

The lookups in DGIdb and PHAROS were performed using the web-based tool. DGIdb assigns an interaction score to the drug–gene interactions, which is the result of combining publication count, source count, relative drug specificity and relative gene specificity. The PHAROS database classifies targets into four ‘Target Development Levels’, according to the evidence of drug interactions available: ‘Tdark’ contains understudied targets, ‘Tbio’ contains highly studied targets but without interaction with compounds, ‘Tchem’ includes targets that bind to small molecules, and ‘Tclin’ interact with approved drugs.

All analyses were done using packages within the R environment (v4.1.2)^76^.

### Reporting summary

Further information on research design is available in the Nature Portfolio Reporting Summary linked to this article.

## Supplementary information

Supplementary Information Supplementary Figs. 1–6

Reporting Summary

Supplementary Tables 1–18

## Data Availability

The GWAS summary data analysed in this study are available from the GIANT (https://portals.broadinstitute.org/collaboration/giant/index.php/GIANT_consortium) and DIAGRAM (https://diagram-consortium.org/) consortia websites, the Open GWAS database (https://gwas.mrcieu.ac.uk/), the GTEx consortium website (https://gtexportal.org/home/ and the MiBioGen repository (https://mibiogen.gcc.rug.nl/). UK Biobank data are available through a procedure described at http://www.ukbiobank.ac.uk/using-the-resource/. Individual-level genetic and clinical data from BioVU cannot be shared publicly due to patient confidentiality. However, summary statistics can be viewed in tabular form at https://phewascatalog.org/labwas/. The DGIdb and the PHAROS databases can be accessed online at https://www.dgidb.org/ and https://pharos.nih.gov/, respectively.

## Code availability

The codes used for our analyses are available at https://github.com/danielcoral/DVA_codes/.
