# Supplementary material for: A phenome-wide comparative analysis of genetic discordance between obesity and type 2 diabetes
Source: Nat Metab. 2023 Jan 26;5(2):237–47. doi: 10.1038/s42255-022-00731-5 (PMC9970876; doi:10.1038/s42255-022-00731-5)

nature metabolism

Article https://doi.org/10.1038/s42255-022-00731-5

A phenome-wide comparative analysis of genetic discordance between obesity and type 2 diabetes

In the format provided by the authors and unedited

Supplementary Figures

Supplementary Figure 1 (Pages 1 – 2): Regional association plots to BMI and T2D for each discordant locus identified. Top strips contain the lead SNP and its nearest gene. A 500 kb window around the lead SNP was used.

Supplementary Figure 2 (Pages 1 – 4): Regional association plots to BMI and T2D for each concordant locus identified. Top strips contain the lead SNP and its nearest gene. A 500 kb window around the lead SNP was used.

Supplementary Figure 3: Results of cell enrichment analyses in DEPICT. Bars represent log10 p-values. Strips in the right show distinct cell lines.

Supplementary Figure 4: Results of tissue enrichment analyses in DEPICT. Bars represent log10 p-values. Strips in the right show distinct tissue categories.

Supplementary Figure 5: Results of systems enrichment analyses in DEPICT. Bars represent log10 p-values. Strips in the right show distinct systems categories.

Supplementary Figure 6: Relationship between BMI estimates and difference between crude and BMI-adjusted estimates for T2D in concordant and discordant SNPs.

Supplementary Figure 1 – Page 1
Locus zoom plots – Discordant profile

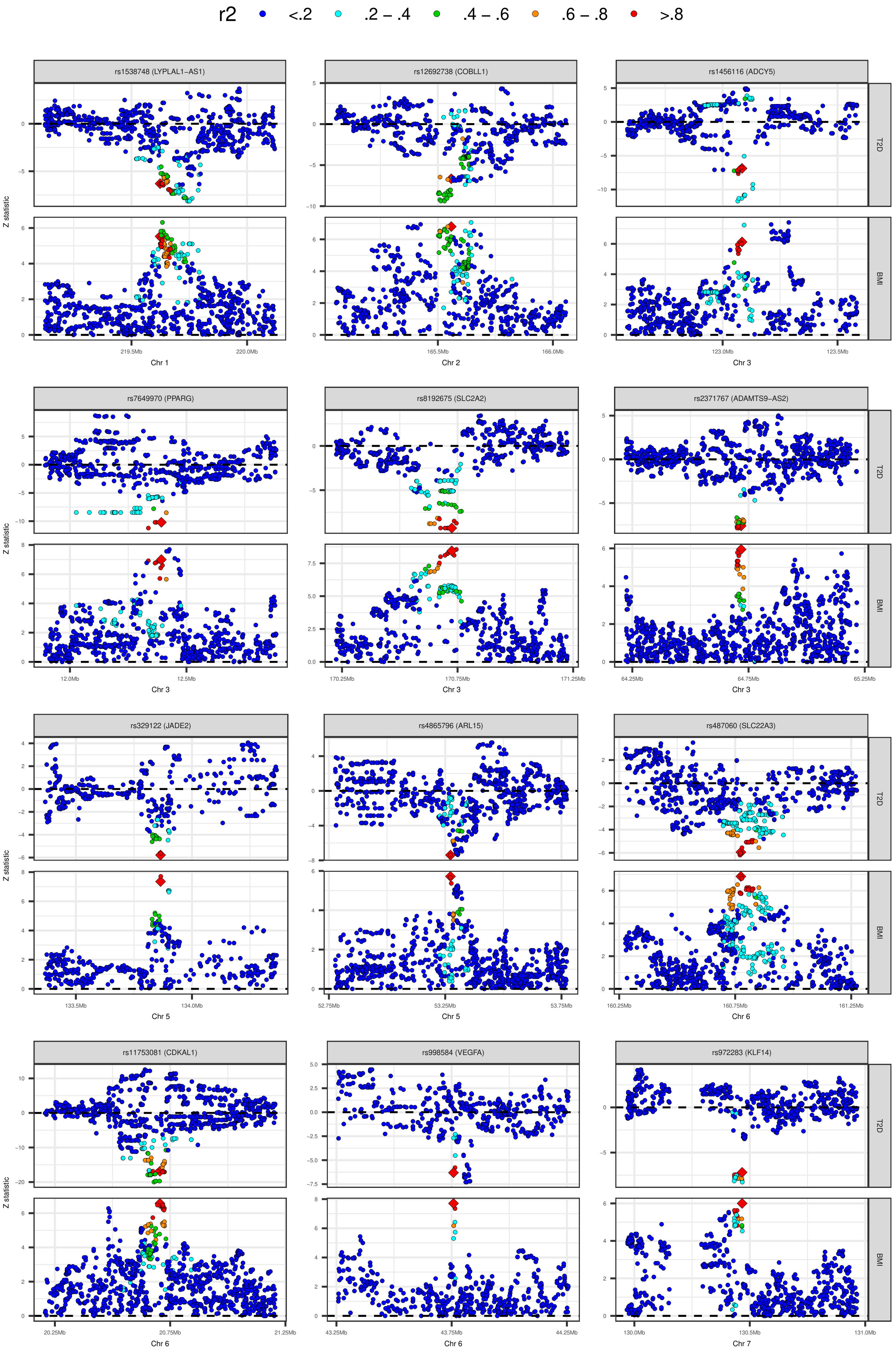

Supplementary Figure 1 – Page 2
Locus zoom plots – Discordant profile

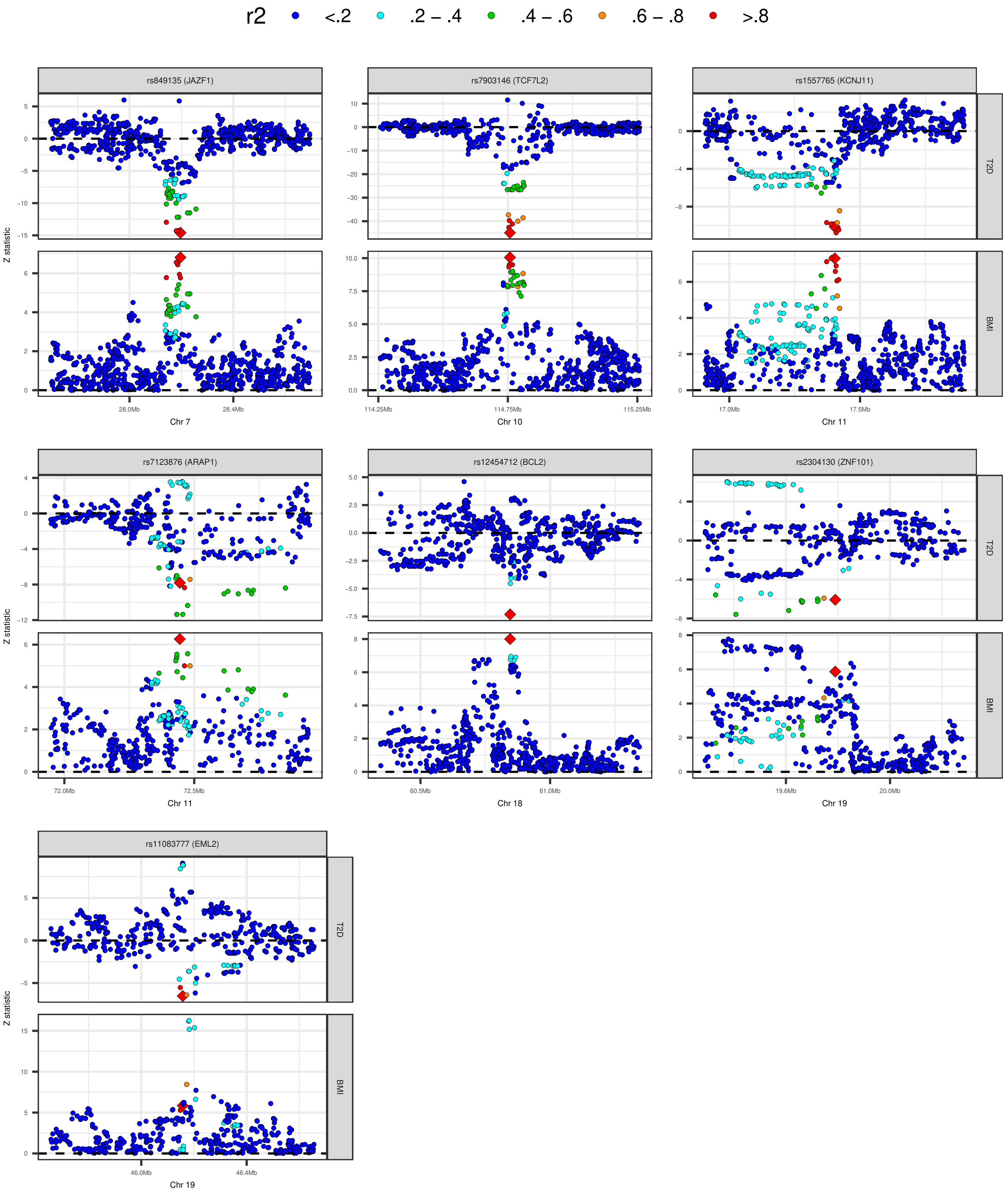

Supplementary Figure 2 – Page 1
Locus zoom plots – Concordant profile

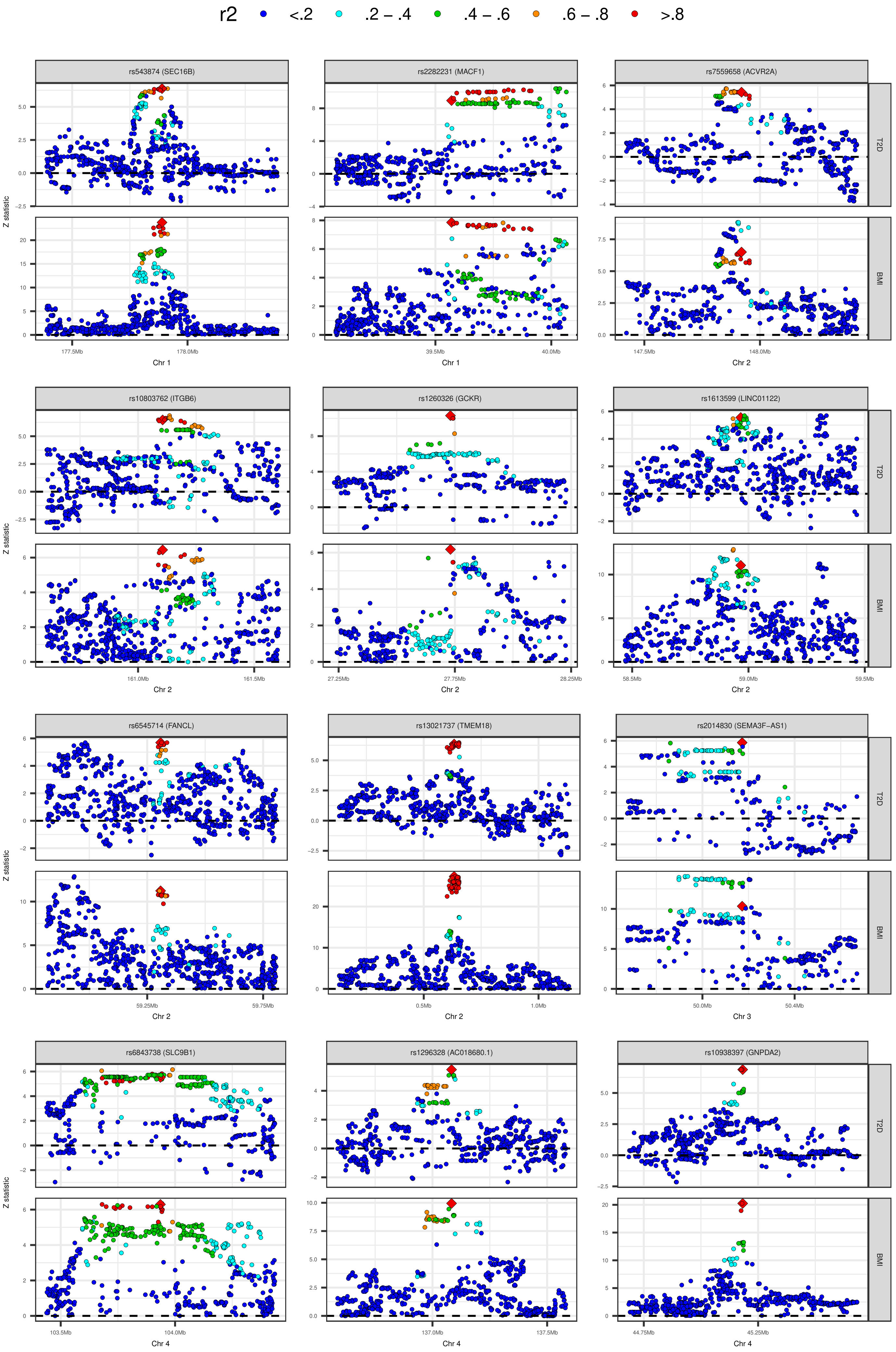

Supplementary Figure 2 – Page 2
Locus zoom plots – Concordant profile

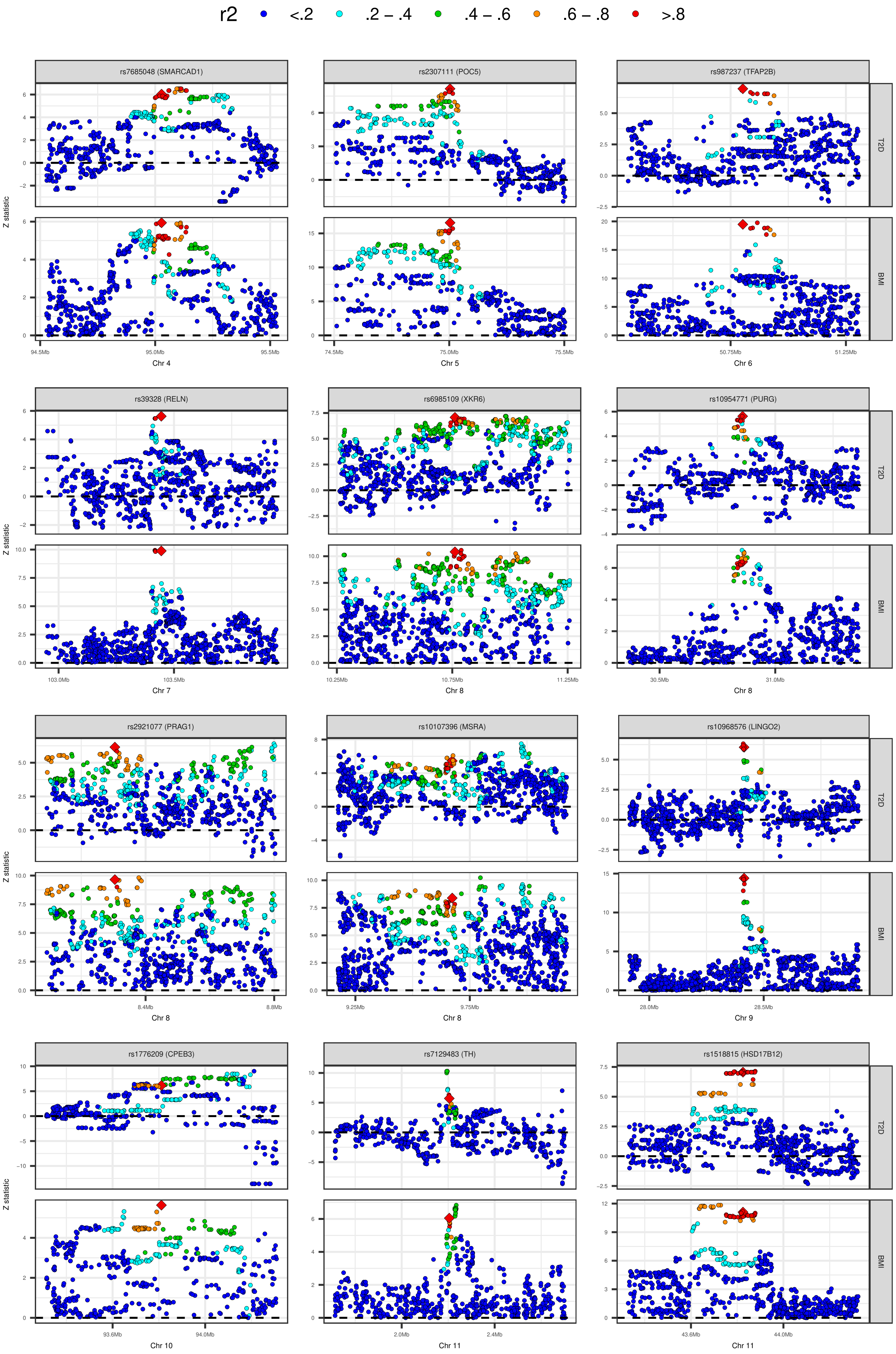

Supplementary Figure 2 – Page 3
Locus zoom plots – Concordant profile

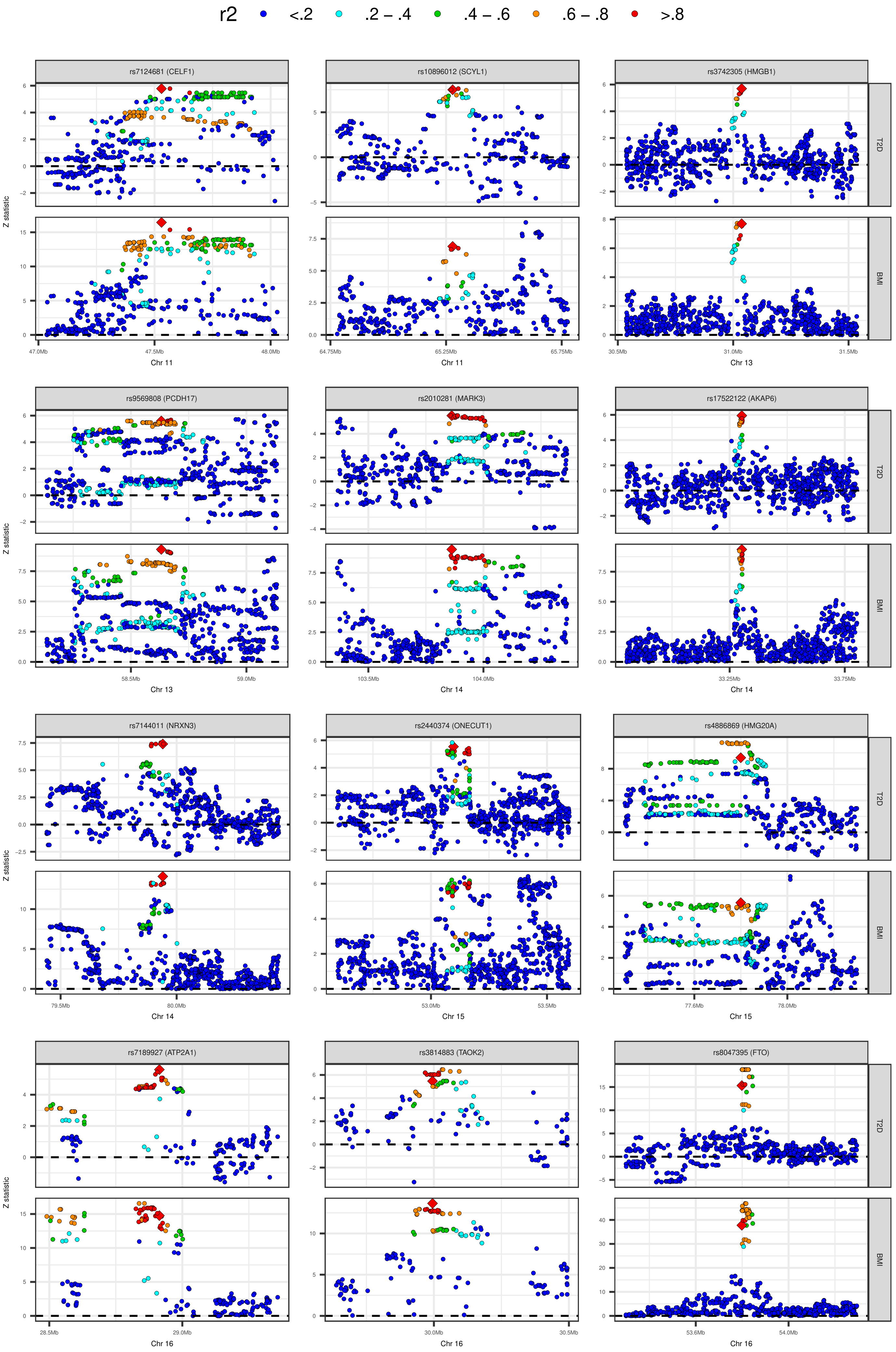

Supplementary Figure 2 – Page 4
Locus zoom plots – Concordant profile

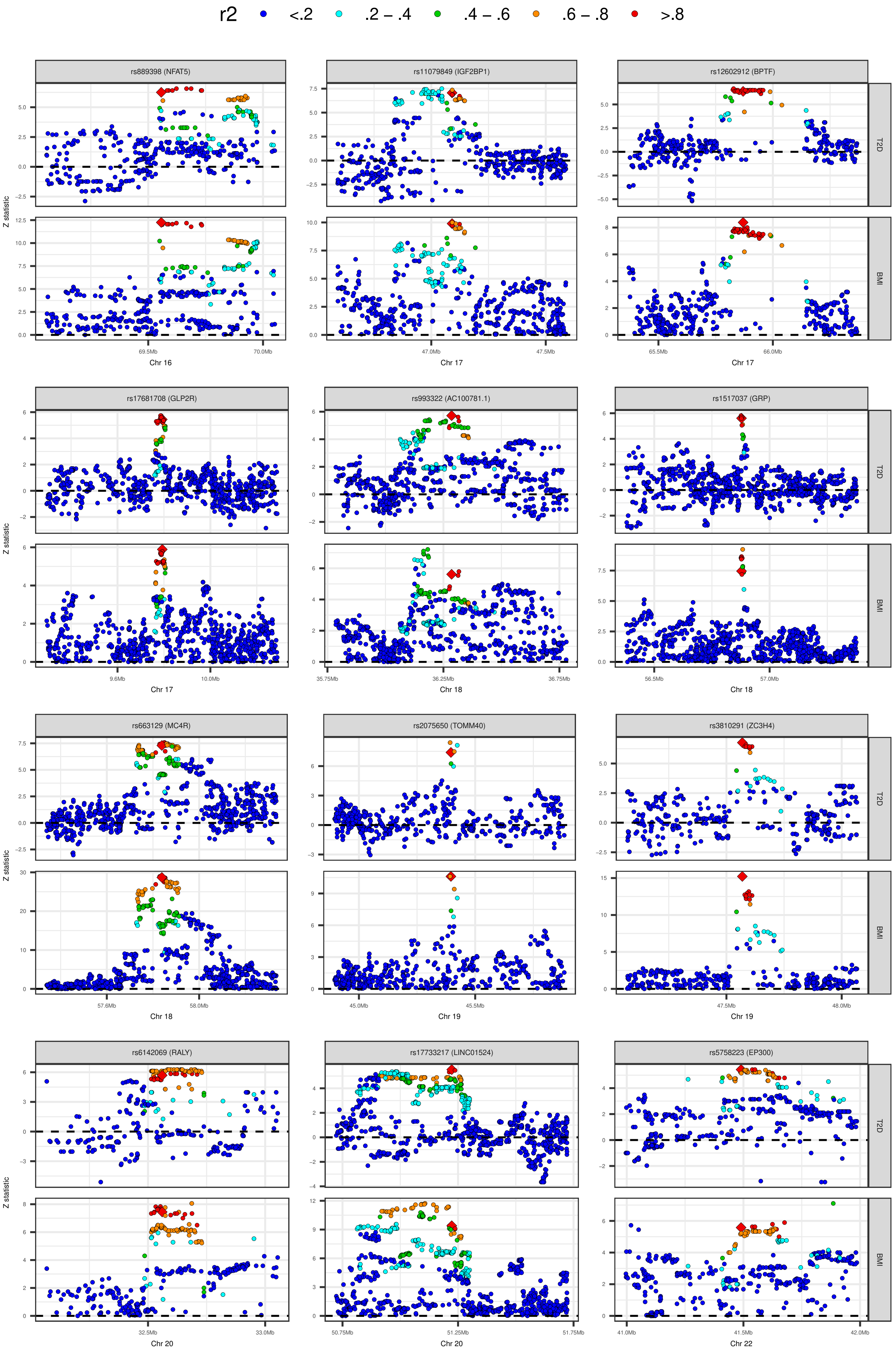

Supplementary Figure 3

Cell enrichment - DEPICT

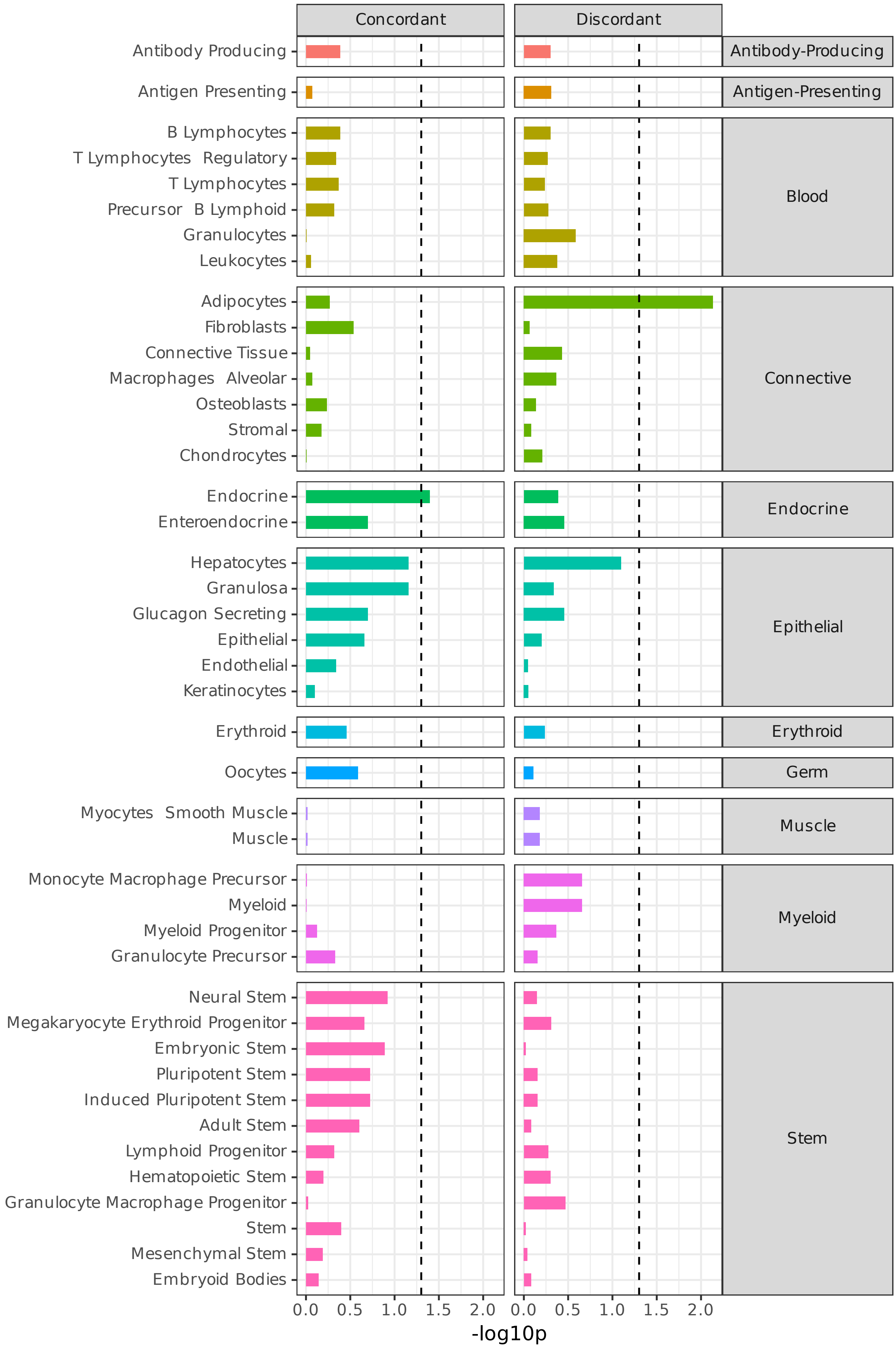

Supplementary Figure 4

Tissue enrichment - DEPICT

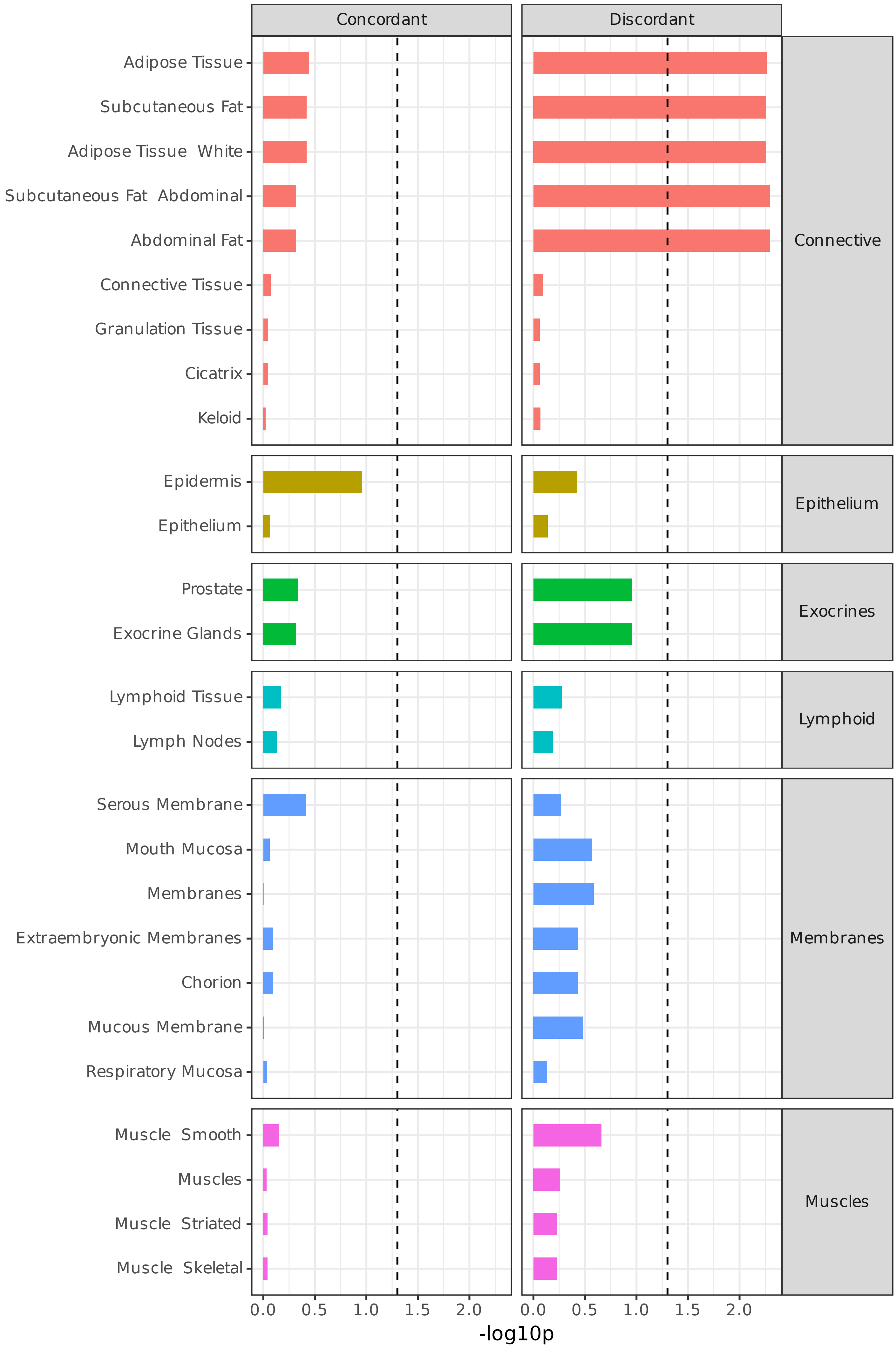

Supplementary Figure 5

Systems enrichment - DEPICT

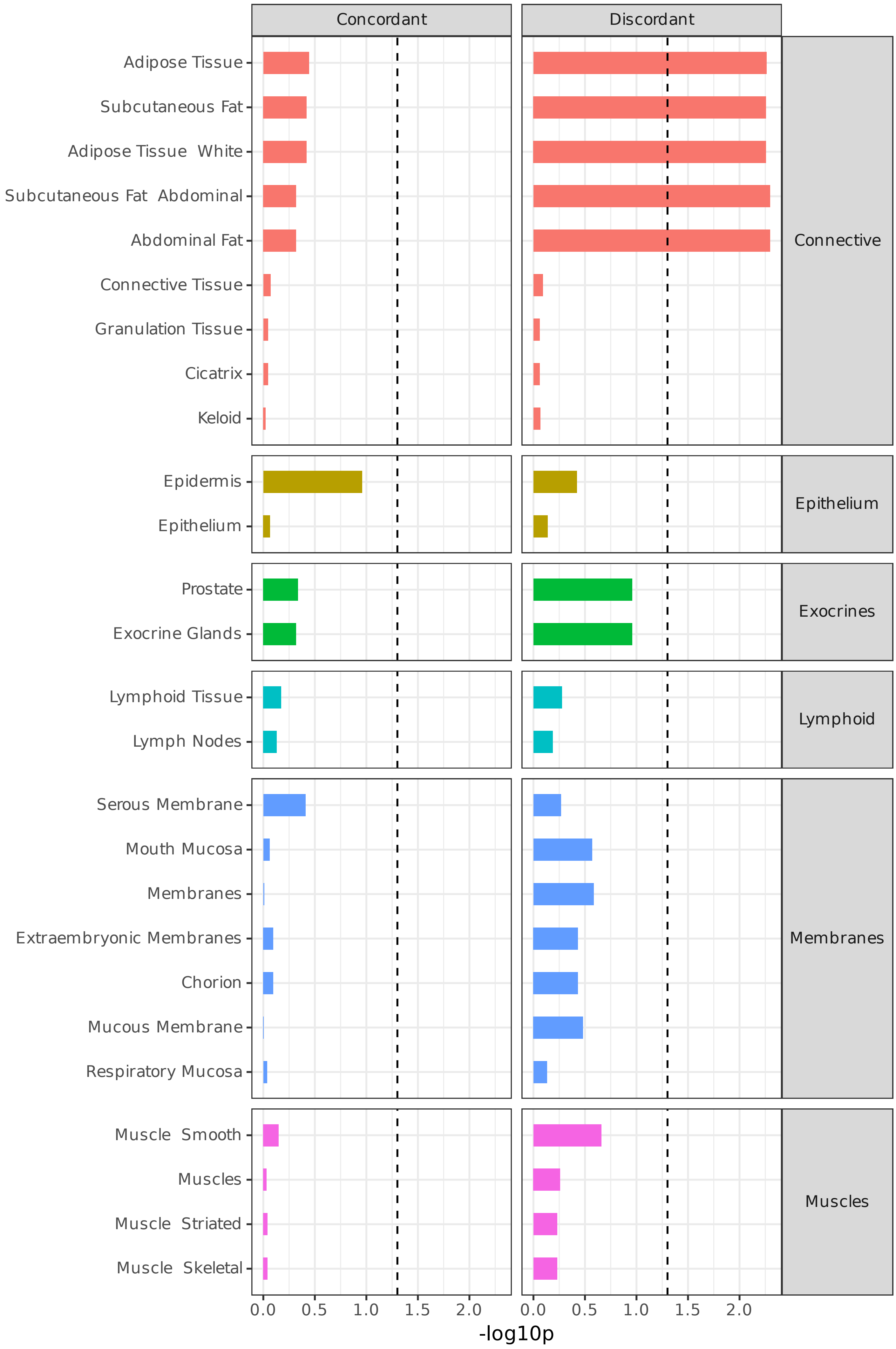

Supplementary Figure 6

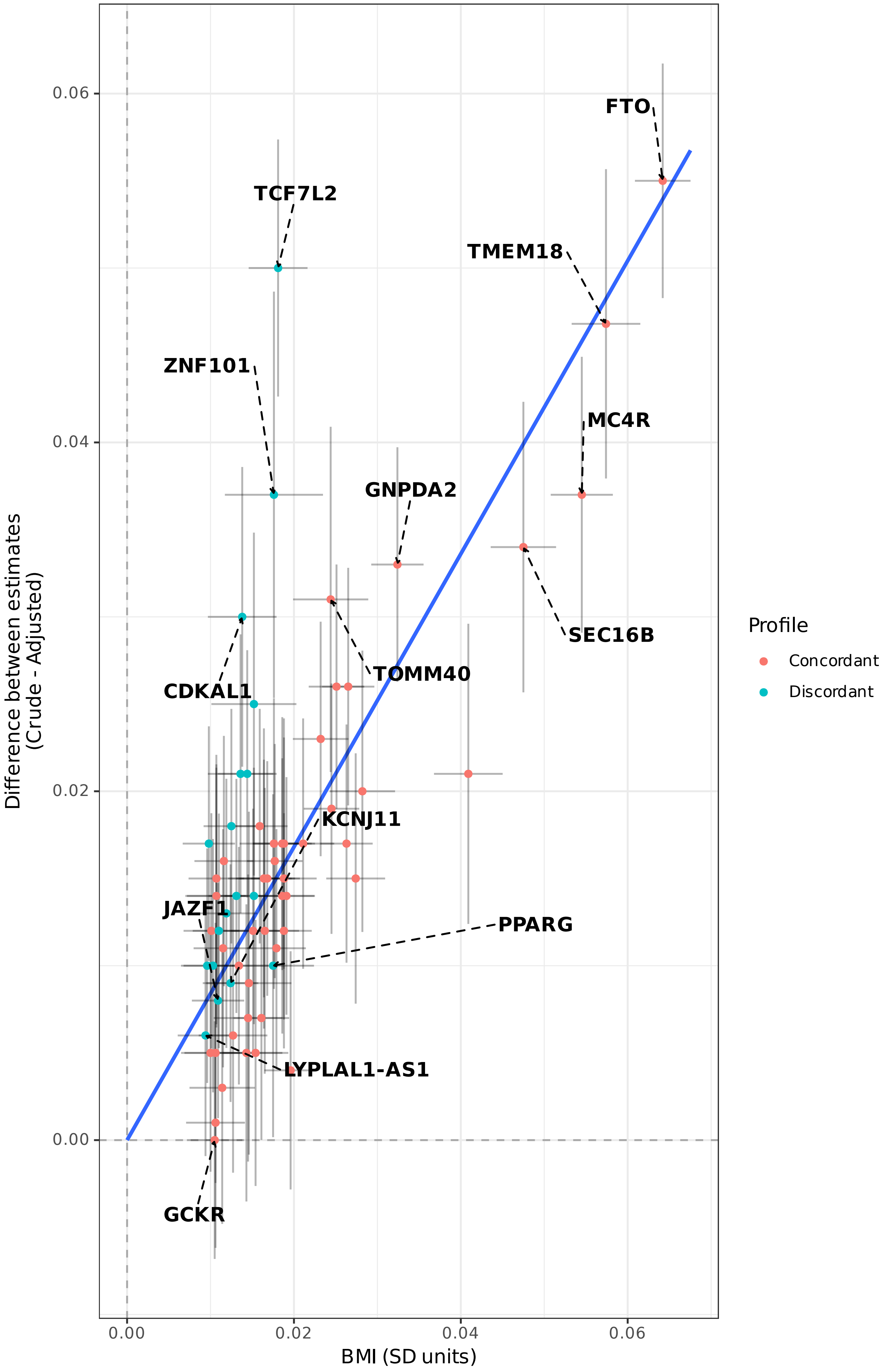

Supplement: Supplementary file 1 — Supplementary Figs. 1–6 [file 42255_2022_731_MOESM1_ESM.pdf]
