# Supplementary material for: A phenome-wide comparative analysis of genetic discordance between obesity and type 2 diabetes
Source: Nat Metab. 2023 Jan 26;5(2):237–47. doi: 10.1038/s42255-022-00731-5 (PMC9970876; doi:10.1038/s42255-022-00731-5)
Supplement: Supplementary file 2 — Reporting Summary [file 42255_2022_731_MOESM2_ESM.pdf]

nature portfolio

Corresponding author(s): Paul W. Franks

Last updated by author(s): Dec 15, 2022

Reporting Summary

Nature Portfolio wishes to improve the reproducibility of the work that we publish. This form provides structure for consistency and transparency in reporting. For further information on Nature Portfolio policies, see our Editorial Policies and the Editorial Policy Checklist.

Statistics

For all statistical analyses, confirm that the following items are present in the figure legend, table legend, main text, or Methods section.

n/a	Confirmed	
☐	☒	The exact sample size (*n*) for each experimental group/condition, given as a discrete number and unit of measurement
☒	☐	A statement on whether measurements were taken from distinct samples or whether the same sample was measured repeatedly
☐	☒	The statistical test(s) used AND whether they are one- or two-sided<br>*Only common tests should be described solely by name; describe more complex techniques in the Methods section.*
☐	☒	A description of all covariates tested
☐	☒	A description of any assumptions or corrections, such as tests of normality and adjustment for multiple comparisons
☐	☒	A full description of the statistical parameters including central tendency (e.g. means) or other basic estimates (e.g. regression coefficient) AND variation (e.g. standard deviation) or associated estimates of uncertainty (e.g. confidence intervals)
☐	☒	For null hypothesis testing, the test statistic (e.g. *F*, *t*, *r*) with confidence intervals, effect sizes, degrees of freedom and *P* value noted<br>*Give P values as exact values whenever suitable.*
☒	☐	For Bayesian analysis, information on the choice of priors and Markov chain Monte Carlo settings
☒	☐	For hierarchical and complex designs, identification of the appropriate level for tests and full reporting of outcomes
☒	☐	Estimates of effect sizes (e.g. Cohen's *d*, Pearson's *r*), indicating how they were calculated

Our web collection on statistics for biologists contains articles on many of the points above.

Software and code

Policy information about availability of computer code

Data collection	We collected GWAS summary statistics for BMI and T2D from the GIANT and the DIAGRAM consortium websites, respectively, using the command line interface. The links are provided in the computer codes. We downloaded data from multiple large consortia from the publicly available repository Open GWAS, hosted by the University of Bristol Medical Research Council Integrative Epidemiology Unit, which we queried through the R interface using the "ieugwasr" package. Summary statistics for tissue specific gene expression and microbiome data were downloaded from the GTEx and MiBioGen online repositories, respectively, using the command line interface, whose links are also provided in the computer code. Epigenetic data generated by the RoadMap Epigenomics Project were collected through the R interface using the "haploR" package. Finally, we queried the genes and proteins with likely pleiotropic effects on BMI and T2D in oppositve directions in the publicly available drug target repositories DGIdb and PHAROS. The R version used was 4.1.2. The codes used for our analyses are available at https://github.com/danielcoral/DVA_codes.
Data analysis	Data manipulation was done using mainly packages from the "tidyverse" collection. Pooled concordant and discordant estimates were calculated using the package "meta". To select traits where we find the most relevant differences between genetically determined obesity profiles we used the package "Boruta". For the comparison in BioVU we used the 'PheWAS' package in R. For the SMR & HEIDI analysis, we adapted code from the original publication by Zhu et al. 2016 to be used within the R environment. For clumping genetic variants we used PLINK version 1.9. The R version used was 4.1.2. The codes used for our analyses are available at https://github.com/danielcoral/DVA_codes.

For manuscripts utilizing custom algorithms or software that are central to the research but not yet described in published literature, software must be made available to editors and reviewers. We strongly encourage code deposition in a community repository (e.g. GitHub). See the Nature Portfolio guidelines for submitting code & software for further information.

Data

Policy information about availability of data

All manuscripts must include a data availability statement. This statement should provide the following information, where applicable:

- Accession codes, unique identifiers, or web links for publicly available datasets
- A description of any restrictions on data availability
- For clinical datasets or third party data, please ensure that the statement adheres to our policy

The GWAS summary data analysed in this study are available from the GIANT (https://portals.broadinstitute.org/collaboration/giant/index.php/GIANT_consortium) and DIAGRAM (https://diagram-consortium.org/) consortia websites, the Open GWAS database (https://gwas.mrcieu.ac.uk/), the GTEx consortium website (https://gtexportal.org/home/ and the MiBioGen repository (https://mibiogen.gcc.rug.nl/). UK Biobank data are available through a procedure described at http://www.ukbiobank.ac.uk/using-the-resource/. Individual level genetic and clinical data from BioVU cannot be shared publicly due to patient confidentiality. However, summary statistics can be viewed in tabular form at: https://phewascatalog.org/labwas. The DGIdb and the PHAROS databases can be accessed online at https://www.dgidb.org/ and https://pharos.nih.gov/, respectively.

Human research participants

Policy information about studies involving human research participants and Sex and Gender in Research.

Reporting on sex and gender	We found that concordant and discordant genetic profiles differ in waist-to-hip ratio, a measure of central to peripheral obesity, predominantly in women. Therefore, when assessing whether individuals with extreme concordant and discordant GRS conveyed obesity profiles that are different from other obesity, we included an additional analysis were we stratified by sex. Sex was determined by genotyping analysis; individuals whose genetic sex did not match reported sex were excluded, in order to have results relevant to biological sex and guard against distortion of estimates due to possible sex chromosome aneuploidies.<br>These and other analyses that we perfomed in UK Biobank only included individuals from European descent. This is because our initial step to find concordant and discordant variants was done using GWAS summary statistics that were done in European populations. European descent was determined using genotyping data.<br>The association of concordant and discordant GRS with laboratory measures were tested on individuals of African descent in BioVU. This was also determined by genotyping data.
Population characteristics	The mean age of individuals in UK Biobank when they attended the first assessment centre was 56 years, ranging between 37 to 85 years. 54% of participants are females.<br>To analyse the association between concordant and discordant GRS to diagnoses in BioVU we included up to 48,544 individuals of European descent. In the analyses of laboratory measures, we included 68,724 participants of European descent and 13,661 participants of African descent. In both the proportion of females is around 51%.
Recruitment	UK Biobank participants were assessed between 2006 and 2010 in 22 assessment centres throughout the UK, covering a variety of different settings to provide socioeconomic and ethnic heterogeneity and urban–rural mix. Invitations to participate were sent via mail to potential participants identified through the National Health Service. Participants that were included are not representative of the sampling population, as there is evidence for healthy volunteer selection bias.<br>Recruitment in BioVU consists of an opt-out clinical collection of patients from the Vanderbilt University Medical Center (VUMC) in an outpatient setting. DNA is extracted from discarded blood drawn for routine clinical care. This is also not representative of the general population of Tennessee and the United States, due to its dependence on clinical registry.
Ethics oversight	Ethics approval for the UK Biobank was obtained from the North West Centre for Research Ethics Committee. Analysis of individual level data from UK Biobank participants in Lund University was approved by the Swedish Ethical Review Authority (2021-0317). The BioVU project was approved by the VUMC Institutional Review Board. The analysis of individual level data was performed in VUMC, and only summary results were shared with researchers at Lund University. Both studies conformed to the ethical principles for medical research involving human participants outlined in the Declaration of Helsinki. All participants provided written informed consent at enrolment.

Note that full information on the approval of the study protocol must also be provided in the manuscript.

Field-specific reporting

Please select the one below that is the best fit for your research. If you are not sure, read the appropriate sections before making your selection.

[x] Life sciences [ ] Behavioural & social sciences [ ] Ecological, evolutionary & environmental sciences

For a reference copy of the document with all sections, see nature.com/documents/nr-reporting-summary-flat.pdf

Life sciences study design

All studies must disclose on these points even when the disclosure is negative.

Sample size	To obtain genetic variants with significant effects on BMI and T2D, we selected studies including >700,000 participants, which were the studies with the largest sample sizes to date for both traits. For the phenome-wide scan, we selected studies with at least 500 individuals since for variants with MAF higher than 10% (the minimum value for concordant and discordant SNP sets, see Supplementary Table 1) and expecting an effect of at least 0.1 standard deviation units the power to detect an association under a nominal alpha value of 0.05 and an additive genetic mode is higher than 80%. In BioVU, we selected diagnoses with at least 200 cases, as recommended in previous simulations of phenome-wide association studies (citation 74 in the manuscript). To assess the association with mortality, we calculated a statistical power of 41% to detect an association between a SNP with a MAF higher than 10% and cardiovascular mortality, expecting a event rate of 1 in every 1000 person-years (using the "survSNP package" in R).
Data exclusions	In the phenome-wide comparison between concordant and discordant SNPs, we excluded studies that were performed in population other than Europeans, to be able to compare effects across phenotypes. To test the association of concordant and discordant GRSs with diseases in BioVU we only included population from European descent. We tested the association of these GRS with laboratory values separately for European and African descent individuals. To test the association with mortality in UK Biobank, we only included individuals of European ancestry. In both BioVU and UK Biobank, we excluded individuals with inconsistency between their reported and genetic sex, had sex chromosome aneuploidy or were outliers for heterozygosity or missingness.
Replication	To verify the sign of the association of concordant and discordant SNPs on T2D we used GWAS summary data from FinnGen. To verify our results of the comparison between concordant and discordant obesity profiles in GWAS summary data, we constructed genetic risk scores with each SNP set and assessed their associations with diseases and laboratory values in an independent dataset (BioVU). After correcting for multiple testing, we checked if the associations reflected the findings from the summary data. We successfully replicated our results. These replication procedures were performed only once.
Randomization	This study does not involve randomization. However, in our analyses we constructed two genetically determined obesity profiles using genetic instruments that are randomly allocated at birth and remain invariant throughout life, which helps prevent confounding and reverse causality. All genetic associations gathered included age and sex as covariates.
Blinding	This study does not involve blinding. GWAS are observational studies and therefore participants know at least the exposure or the outcome, and often both.However, by using genetic instruments randomly allocated we prevent confounding and reverse causality in our analyses.

Reporting for specific materials, systems and methods

We require information from authors about some types of materials, experimental systems and methods used in many studies. Here, indicate whether each material, system or method listed is relevant to your study. If you are not sure if a list item applies to your research, read the appropriate section before selecting a response.

Materials & experimental systems

n/a	Involved in the study	
☒	☐	Antibodies
☒	☐	Eukaryotic cell lines
☒	☐	Palaeontology and archaeology
☒	☐	Animals and other organisms
☒	☐	Clinical data
☒	☐	Dual use research of concern

Methods

n/a	Involved in the study	
☒	☐	ChIP-seq
☒	☐	Flow cytometry
☒	☐	MRI-based neuroimaging
